# Molecular insights into the substrate recognition and quasi-unidirectional catalysis of OpaE, an atypical fungal enzyme from the Asp/Glu racemase family

**DOI:** 10.1016/j.jbc.2025.110828

**Published:** 2025-10-16

**Authors:** Xuehua Zheng, Zhiyong Guo, Leyao Chen, Haowen Wang, Johannes Freitag, Aitao Li, Gert Bange, Shu-Ming Li, Liujuan Zheng

**Affiliations:** 1 Center for Synthetic Microbiology (SYNMIKRO) & Department of Chemistry, Marburg University, Marburg, Germany; 2 Guangzhou Municipal and Guangdong Provincial Key Laboratory of Molecular Target & Clinical Pharmacology, the NMPA and State Key Laboratory of Respiratory Disease, School of Pharmaceutical Sciences, Guangzhou Medical University, Guangzhou, China; 3 State Key Laboratory of Biocatalysis and Enzyme Engineering, Hubei Key Laboratory of Industrial Biotechnology, School of Life Sciences, Hubei University, Wuhan, China; 4 Department of Pharmacy, Institute of Pharmaceutical Biology and Biotechnology, Marburg University, Marburg, Germany; 5 Max-Planck Institute for Terrestrial Microbiology, Molecular Physiology of Microbes, Marburg, Germany

**Keywords:** Asp/Glu racemase superfamily, OpaE, “quasi-unidirectional” catalysis, X-ray crystallography, substrate specificity, enzyme evolution

## Abstract

Enzymes of the Asp/Glu racemase family, mostly found in bacteria, typically catalyze bidirectional reactions with similar rate because of their pseudosymmetrical structure and minimal differences between substrate isomers. We recently identified OpaE, an atypical fungal enzyme that catalyzes the d-to-l conversion of nonribosomal peptide derivatives in oxepinamide E/F biosynthesis from *Aspergillus ustus*. However, the structure and catalytic mechanism remained poorly understood. In this study, we present the crystal structure of OpaE, which reveals a pseudosymmetrical architecture characteristic of bidirectional racemases, yet it catalyzes the “quasi-unidirectional” d-to-l conversion, yielding approximately 90% of the l-form. This “quasi-unidirectional” activity arises from structural divergence between substrate isomers, which leads to differences in binding modes, energy barriers, and intermediate stabilities, as well as distinct thermodynamic properties, as revealed by crystal structure, docking, molecular dynamics, and quantum mechanics/molecular mechanics–based metadynamics simulations. Phylogenetic analysis revealed that OpaE has evolved specific structural adaptations, including hydrophobic pockets and loop shifts, to optimize substrate binding and catalysis, which ultimately favor “quasi-unidirectional” activity and reflect a distinct evolutionary path. In summary, our study deepens our understanding of the structural and mechanistic diversity within the Asp/Glu family and highlights OpaE's potential for biotechnological applications, particularly in chiral substrate transformations.

In nonribosomal peptide (NRP) biosynthesis, the interconversion between l- and d-amino acids plays a critical role in the assembly process and determines the biological activity of the final products (1). This process is typically catalyzed by the epimerase domain (E domain) of the megaenzymes, nonribosomal peptide synthetases (NRPSs), which usually facilitates the conversion from l- to d-amino acids during peptide elongation (2). In our previous study, we elucidated the biosynthetic pathway of oxepinamides E and F in *Aspergillus ustus*, two tricyclic peptide derivatives with an oxepine moiety and exhibiting potential therapeutic applications in Alzheimer’s disease, atherosclerosis, diabetes, and inflammation (3, 4). The peptide backbone of oxepinamides E/F is synthesized by the NRPS OpaA, whose E domain converts l-Phe to d-Phe during NRP assembly. Remarkably, the tailoring enzyme OpaE catalyzes the re-epimerization of this d-Phe residue, originally introduced by OpaA, back to the l-configuration (Fig. 1*A*), a critical step that enables subsequent methylation by OpaF (3).

**Figure 1:**
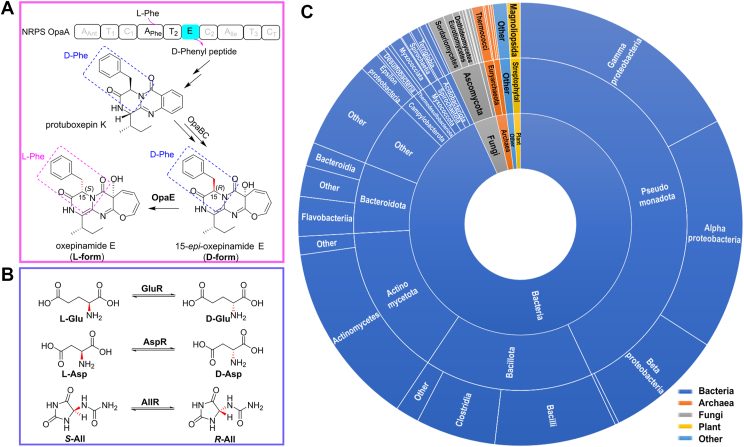
**Catalytic reactions and distribution of Asp/Glu racemase family enzymes.***A*, epimerization role of Asp/Glu racemase OpaE in the fungal biosynthesis pathway. *B*, representative reactions catalyzed by bacterial Asp/Glu racemases. The reactions catalyzed by representative bacterial Asp/Glu racemase, including glutamate racemase (GluR), aspartate racemase (AspR), and allantoin racemase (AllR), are shown in a *blue box*. *C*, distribution of Asp/Glu racemase family enzymes. The distribution of Asp/Glu racemase family enzymes (PF01177) is based on 75,000 sequences from the UniProt database (accessed on January 25, 2025). Among these, the vast majority (69,389) are from bacteria, which are widely distributed across multiple phyla, including Pseudomonadota (gamma-, alpha-, and beta-proteobacteria), Bacillota (Bacilli and Clostridia), Actinomycetota, and Bacteroidota. In stark contrast, only 2348 sequences are from fungi.

Bioinformatics analysis indicates that OpaE belongs to the Asp/Glu racemase superfamily (PF01177) as classified in the UniProt database (5). This superfamily is known for catalyzing the stereochemical interconversion of acidic amino acids, including glutamate and aspartate, as well as nitrogen-containing compounds, such as hydantoin and aryl malonate (Fig. 1*B*) (6, 7, 8, 9). These enzymes play an essential role in producing d-glutamate, a critical precursor for the biosynthesis of peptidoglycan, the structural backbone of bacterial cell walls (7). Reflecting this crucial function, orthologous Asp/Glu racemase genes are widely distributed across most bacterial species. In contrast, eukaryotic organisms, including fungi, lack peptidoglycan-based cell walls and do not require d-glutamate for structural integrity. This divergence likely explains the relatively small number of fungal sequences in the Asp/Glu racemase superfamily, with only 2348 entries out of 74,000 in the UniProt database (Fig. 1*C*). OpaE is the only well-characterized member in fungi. Its ability to process a complex tricyclic substrate, which is distinct from the simpler substrates typically handled by bacterial enzymes, suggests unique structural adaptations.

In this study, we present the crystal structure of OpaE, offering structural insights into a fungal enzyme from the Asp/Glu racemase superfamily. Unlike high activities in both reaction directions commonly observed in bacterial homologs, OpaE predominantly favors the conversion from d-to-l form (*R*- to *S*-configuration) of the phenylalanyl residue (over 90%). To better describe this predominant catalytic characteristic, the term “quasi-unidirectional” is utilized in this study. Through a combination of mutagenesis, docking, and quantum mechanics/molecular mechanics (QM/MM) metadynamics simulations, we elucidate the molecular mechanisms underlying OpaE's catalytic activity and substrate specificity. Evolutionary analysis reveals its catalytic specialization within fungal secondary metabolism. These findings not only broaden our understanding of Asp/Glu racemases but also provide a structural foundation for biotechnological applications in peptide synthesis and modification of small molecules, including natural products.

## Results

### OpaE catalyzes the “quasi-unidirectional” conversion of 15-*epi*-oxepinamide E (d-form) to oxepinamide E (l-form)

To investigate the reversibility of OpaE's catalytic activity, we systematically investigated its catalytic properties using both d-form (15-*epi*-oxepinamide E) and l-form (oxepinamide E) as substrates, monitoring their interconversion under varying enzyme and substrate concentrations (Table 1 and Fig. S1). With 1 mM d-form as the substrate, l-form production increased from 60% to over 90% as OpaE concentration rose from 1.3 μM to 5.2 μM. In contrast, using 1 mM l-form as the substrate resulted in less than 10% conversion to d-form, even at higher enzyme concentrations, up to 11.5 μM. We also tested substrate concentrations ranging from 0.5 mM to 2 mM at a fixed enzyme concentration of 5.2 μM. Notably, 91% of the d-form was converted to the l-form, whereas less than 10% of the l-form was converted to the d-form under these conditions. These results suggest that OpaE strongly favors the conversion of d-form to l-form, with minimal influence from variations in substrate concentration.

**Table 1:** Reaction profiles for d-to-l/l-to-d conversions at varying substrate and enzyme concentrations

Substrate (mM)	Enzyme (μM)	Reaction type	Product	
			l-form (%)	d-form (%)
1	1.3	d-to-l	59.7 ± 1.7	
		l-to-d		8.3 ± 0.1
1	5.2	d-to-l	91.8 ± 0.4	
		l-to-d		8.3 ± 0.1
1	11.5	d-to-l	90.7 ± 0.1	
		l-to-d		7.8 ± 0.1
0.5	5.2	d-to-l	92.1 ± 0.1	
		l-to-d		7.9 ± 0.1
2	5.2	d-to-l	91.5 ± 0.9	
		l-to-d		9.2 ± 0.1

d-form and l-form refer to 15-*epi*-oxepinamide E and oxepinamide E, respectively.

### Structural insights into OpaE revealed the catalytic cysteine dyad

**Figure 2:**
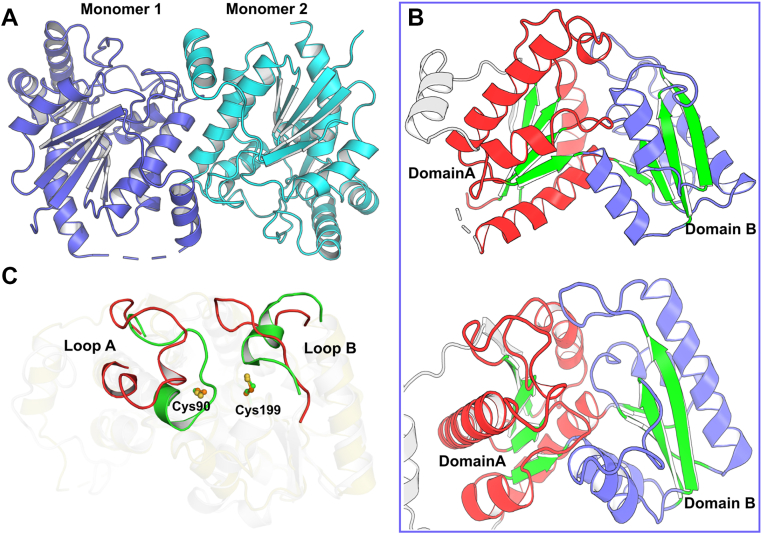
**Crystal structure of OpaE and comparison with homologous enzyme *Kp*AllR.***A*, overall crystal structure of the OpaE dimer (PDB ID: 9QBX), with one monomer displayed in *cyan* and the other in *tv-blue*. *B*, detailed view of a single monomer, highlighting its two symmetrical domains. The β-sheets are highlighted in *green*, whereas the α-helices are shown in *red* and *tv-blue*, respectively. *C*, structural alignment of OpaE with *Kp*AllR (PDB ID: 3QVJ). OpaE is shown in *orange*, and *Kp*AllR is shown in *gray*, with the catalytic cysteine residues and displaced loop regions highlighted in *tv-red* and *green* in OpaE and *Kp*AllR, respectively.

To understand the structural basis of OpaE catalysis, we determined its crystal structure at 2.65 Å resolution (Protein Data Bank [PDB] ID: 9QBX). The structure revealed a homodimeric assembly (Fig. 2*A*), with each monomer comprising two domains arranged in pseudo-twofold symmetry (Fig. 2*B*), a hallmark of the Asp/Glu racemase superfamily. Each domain contains a four-stranded parallel β-sheet flanked by two pairs of α-helices, forming a compact and stable fold essential for catalytic function (Fig. 2*B*). DALI structural comparisons indicate that while OpaE retains the conserved Asp/Glu racemase fold, its overall similarity to related enzymes is moderate. Structural alignment with *Klebsiella pneumoniae* allantoin racemase (*Kp*AllR, PDB ID: 3QVJ) shows an RMSD of 2.6 Å and an Z-score of 22.1, whereas alignment with *Pseudomonas fluorescens* allantoin racemase (PDB ID: 5LG5) results in an RMSD of 2.9 Å and an Z-score of 21.9 (Table S2). Notably, despite the well-aligned core β-sheets, OpaE exhibits significant displacement in the loop regions and adjacent helices, particularly in loops A and B (Fig. 2*C*), which leads to an expanded substrate-binding pocket. This expansion may enhance the enzyme’s ability to accommodate larger or more complex substrates, such as 15-*epi*-oxepinamide E.

**Figure 3:**
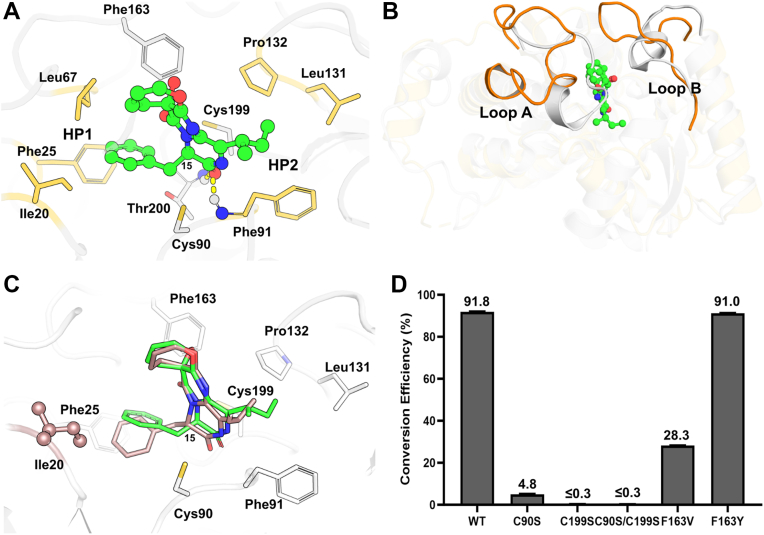
**Predicted substrate binding and structural adaptations in OpaE.***A*, predicted binding mode of 15-*epi*-oxepinamide E (d-form) in OpaE. The substrate is shown in *green*, and the residues in HP1 and HP2 pockets are highlighted in *yellow*. *B*, structural alignment of substrate-bound OpaE with *Kp*AllR. OpaE is represented in *orange*, and *Kp*AllR is represented in *gray*. *C*, docking simulations of oxepinamide E (l-form) in OpaE. l-form, along with the potential clash with residue Ile20, is shown in *dark salmon*, whereas d-form is colored *green* for comparison. *D*, epimerization activity of OpaE mutations toward 15-*epi*-oxepinamide E. All assays were performed using 1 mM substrate with 5.2 μmol of enzyme.

*Kp*AllR has been reported to catalyze allantoin racemization using two conserved cysteines (Cys79 and Cys184) as catalytic residues (10). Sequence alignments reveal that these cysteines are conserved across the superfamily, corresponding to Cys90 and Cys199 in OpaE (Fig. S2). Structural superimposition of OpaE with *Kp*AllR shows that Cys90 and Cys199 in OpaE align well with Cys79 and Cys184 in *Kp*AllR, positioning them at the interface of the two domains (Fig. 2*C*). In addition, a distinct electron density feature was observed between Cys90 and Cys199 (Fig. S3*A*), suggesting a potential substrate-binding site. These structural features suggest that Cys90 and Cys199 in OpaE may serve as the catalytic residues. To validate this hypothesis, we generated the C90S, C199S, and C90S/C199S mutants of OpaE. Activity assays revealed that the OpaE_C90S mutant exhibited a significant reduction in epimerization efficiency of 15-*epi*-oxepinamide E to oxepinamide E, retaining only 4.8 ± 0.4% of wildtype activity, whereas the C199S and C90S/C199S mutants almost completely lost activity (≤0.3%), confirming that both cysteines are essential for catalysis (Fig. 3*D*). These findings establish Cys90 and Cys199 as the catalytic dyad of OpaE.

### Substrate binding and active site adaptation revealed by docking and molecular dynamics studies

**Figure 4:**
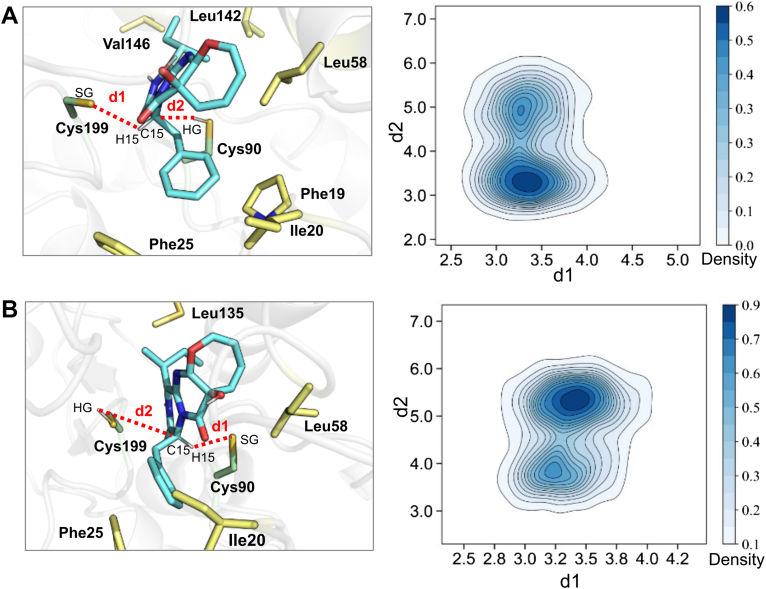
**Representative snapshots and catalytic distance distributions during MD simulations of OpaE–substrate complex.***A*, representative snapshot of d-isomer (*left panel*), along with its two-dimensional density map (*right panel*) showing the distribution of catalytic distances. *B*, representative snapshot of l-isomer (*left panel*), along with its two-dimensional density map (*right panel*) showing the distribution of catalytic distances. d1 represents the distance between the SG atom of the deprotonated cysteine residue and the H15 atom of the substrate, whereas d2 represents the distance between the HG atom of the protonated cysteine residue and the C15 atom of the substrate.

To investigate how substrates bind to OpaE, we initially attempted cocrystallization of both wildtype and mutant enzymes with 15-*epi*-oxepinamide E (d-form) and oxepinamide E (l-form). However, we were unable to obtain crystals of the enzyme–substrate complex. As an alternative, we employed docking and molecular dynamics (MD) simulations to predict the substrate-binding mode. The substrate adopts a T-shaped structure with a fused oxepine–pyrimidinone–ketopiperazine tricyclic backbone and phenylalanine- and isoleucine-derived substituents at the para position on the oxepine ring (Fig. 1*C*). AutoDock simulations suggested a reasonable binding mode for the d-form (Fig. 3*A*), where the phenylalanine side chain fits into hydrophobic pocket 1 (HP1, formed by Ile20, Phe25, and Leu167), and the isoleucine side chain aligns with hydrophobic pocket 2 (HP2, formed by Phe91, Leu131, and Phe132). This binding is stabilized by hydrogen bonds with the backbone nitrogen atoms of Phe91 and Thr200 in the oxyanion hole, as observed in *Kp*AllRs (10), along with strong hydrophobic interactions and edge-to-face stacking with Phe25 within the HPs. MD simulations further supported this binding mode, showing a high proportion of prereaction states with favorable catalytic distances between C-15 or H-15 of the substrate and Cys90–Cys199 in OpaE (Fig. 4*A*). Specifically, both d1 (the distance between the SG atom of deprotonated Cys199 and the H15 atom of the substrate) and d2 (the distance between the HG atom of protonated Cys90 and the C15 atom of the substrate) are less than 3.5 Å.

Furthermore, docking results highlight the crucial role of loop displacement, particularly in loop A (Ala49–Ala59) and loop B (Asn160–Met170), in expanding the active site to accommodate bulkier substrates. Structural superimposition of substrate-bound OpaE with *Kp*AllR reveals that, without the displacement of these loop regions, steric clashes would occur (Fig. 3*B*), emphasizing the necessity of spatial expansion for effective substrate binding. This is further supported by the Phe163 to Val mutation, which markedly reduces activity—from 91.8 ± 0.1% to 28.3 ± 0.2% with 15-*epi*-oxepinamide E and from 8.2 ± 0.2% to 4.8 ± 0.6% with oxepinamide E as substrates (Figs. 3*D*, and S4). In contrast, mutation to tyrosine has no impact on activity (Figs. 3*D*, and S4), highlighting the importance of loop flexibility for substrate accommodation. In addition, OpaE's active site is notably more hydrophobic, with residues such as Ile20, Phe25, Leu131, and Pro132. In contrast, *Kp*AllR contains more polar residues in the corresponding positions, such as Asn12, Met17, Thr118, and Thr119 (Fig. S3*B*). Notably, Thr118 and Thr119 form a polar binding pocket that interacts with the ureido tail of allantoin, helping to control the orientation of the hydantoin ring (6). The increased hydrophobicity in OpaE likely reflects an adaptation to better accommodate the hydrophobic substituents of its substrate, optimizing binding interactions.

To explore the reasons for OpaE's “quasi-unidirectional” catalysis, we also docked the l-form into the OpaE active site (Fig. 3*C*, *dark salmon*). Similar to the d-form (Fig. 3*C*, *green*), the phenylalanyl and isoleucine side chains of oxepinamide E occupy the HPs HP1 and HP2, respectively. However, in this configuration, the H-15 of oxepinamide E faces Cys90, and steric clashes with Ile20 are observed, suggesting reduced catalytic feasibility. MD simulations revealed that while d1 remains below 3.5 Å, d2 predominantly exceeds 5.0 Å, indicating an unfavorable catalytic conformation (Fig. 4*B*). These findings provide insight into OpaE's preference for catalyzing the d-to-l conversion.

### Mechanistic insights into “quasi-unidirectionality” from QM/MM metadynamics studies

**Figure 5:**
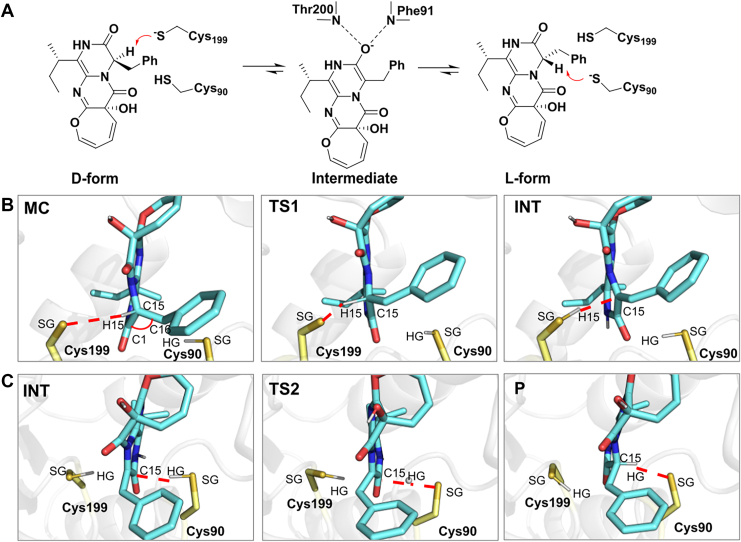
**QM/MM metadynamics calculations of****d****-to-****l****conversion catalyzed by OpaE *via* a stepwise mechanism.***A*, proposed mechanism of racemization catalyzed by OpaE. *B*, active site structures during the first step of the catalytic pathway, showing the Michaelis complex (MC), the transition state of the first reaction step (TS1), and the reaction intermediate (INT). *C*, active site structures during the second step of the catalytic pathway, showing the reaction INT, the transition state of the second reaction step (TS2), and the final product (P). For clarity, hydrogen atoms attached to carbon atoms have been omitted. *Red dashed lines* indicate bond formation and cleavage events between the catalytic Cys residue and the substrate.

**Figure 6:**
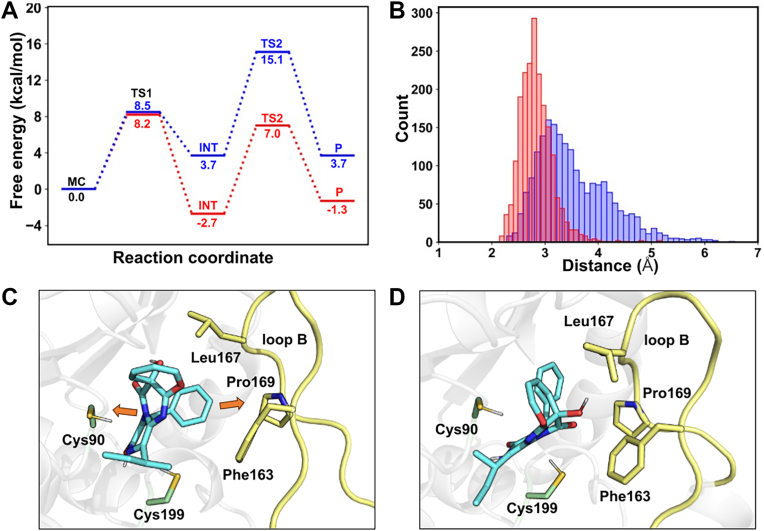
**Free energy profile, catalytic distance distribution, and substrate–loop interactions in OpaE-catalyzed racemization.***A*, free energy profile of the reaction catalyzed by OpaE, derived from QM/MM metadynamics simulations. The free energy changes for the d-to-l conversion are represented by the *red line*, whereas the free energy changes for the l- to d-conversion are shown by the *blue line*. *B*, distance distribution between the catalytic cysteine HG and substrate C15 during MD simulations of the intermediate (INT) structure. The distributions for the d-to-l andl-to-d conversions are colored *red* and *blue*, respectively. *C*, interaction between the substrate and loop B in the INT structure of the l-isomer. *D*, interaction between the substrate and loop B in the INT structure of the d-isomer.

To further investigate the molecular basis for OpaE's “quasi-unidirectional” activity, we performed QM/MM metadynamics simulations to examine the conversion of the d-form (15-*epi*-oxepinamide E) to the l-form (oxepinamide E). We then compared this pathway with the reverse l-to-d conversion to elucidate key differences in their catalytic mechanisms. Representative snapshots from the MD simulation trajectory were selected, with the QM region including the substrate and the two catalytic cysteine residues (Cys90 and Cys199). The stereochemical conversion was found to proceed through a 1,1-proton transfer mechanism in a stepwise manner (Fig. 5*A*), akin to the mechanism observed in the homologous enzyme *Kp*AllR (11). In the first step, the catalytic cysteine abstracts a proton from the C-15 of substrates, forming an intermediate (INT). In the second step, the other catalytic cysteine donates a proton to the INT's C-15 atom, completing the reaction. During the reaction, two transition states were identified: the first (TS1) connects the Michaelis complex (MC, the enzyme–substrate complex in the reactant state) to the INT in the first step, whereas the second (TS2) connects INT to the product (P) in the second step. The active site structures of these states are shown in Figures 5, *B*, *C* and S5, with the corresponding energy change and proposed catalytic mechanism in Figure 6*A*, and the collective variables (CV) used to monitor the proton transfers in Fig. S6 (as described in detail below).

The d-to-l form conversion begins with the SG atom of Cys199 approaching the substrate, with a free energy barrier of 8.2 kcal/mol and an exothermic release of 2.7 kcal/mol in the first step (Figs. 5*A* and 6*A*, *red*). From MC to TS1, the distance between the Cys199-SG and H15 of 15-*epi*-oxepinamide E decreases from 2.91 ± 0.08 Å to 1.80 ± 0.07 Å, whereas the C15-H bond length increases from 1.12 ± 0.06 Å to 1.34 ± 0.08 Å, and the C1–C15–C1′ bond angle shifts from 108 ± 3° to 112 ± 3°. These changes suggest that proton transfer is not yet complete at TS1. As the reaction progresses to INT, the proton is fully transferred to Cys199, as indicated by the SG–H15 distance of 1.40 ± 0.07 Å, the C15–H15 bond length of 2.29 ± 0.06 Å, and the C1–C15–C1' bond angle increasing to 119 ± 2°, which prepares the system for the second reaction step. This step begins with the HG atom of Cys90 approaching the substrate’s C15 atom and has a higher energy barrier of 9.7 kcal/mol, indicating that it is rate limiting and controls the overall reaction rate (Figs. 5*B* and 6*A*, *red*). The reaction proceeds through TS2, where the SG–HG distance is 1.80 ± 0.23 Å and the C15–HG distance is 1.60 ± 0.21 Å, ultimately reaching the product state (SG–HG = 2.60 ± 0.06 Å; C15–HG = 1.12 ± 0.06 Å). Overall, the d-to-l conversion has a thermodynamic advantage, with an exothermic release of 1.3 kcal/mol, favoring the l-form over the d-isomer.

On the other hand, the l-to d-form conversion follows a similar stepwise mechanism to the d-to-l form conversion, but with proton transfer occurring in the opposite direction (Fig. S5). In this pathway, Cys90 abstracts a proton from the oxepinamide E, whereas Cys199 donates a proton to the INT. The energy barrier for the rate-limiting second step (11.4 kcal/mol) is significantly higher than that of the d-to-l form conversion, with a 1.7 kcal/mol difference, whereas the first step has a comparable energy barrier of 8.5 kcal/mol (Fig. 6*A*, *blue*). MD simulations of the INT state reveal that the HG of Cys199 deviates more frequently from the optimal range for transfer to the substrate C15 (Fig. 6*B*). This deviation arises because the phenylalanyl substituent is positioned closer to Pro169 in loop B, creating spatial constraints that force the substrate to bend away from it. This bending increases the distance between Cys199, located beneath loop B, and C15, and makes proton transfer more difficult by disrupting the optimal alignment required for efficient transfer (Fig. 6*C*). In contrast, in the d-to-l form pathway, the INT state remains more stable, with fewer spatial constraints and proton transfer distances more frequently within the optimal range (Fig. 6, *B* and *D*). In addition, the l- to d-form reaction is highly endothermic, with the product state being 3.7 kcal/mol higher in energy than the reactant MC state. These findings highlight that the “quasi-unidirectional” nature of OpaE is driven by both kinetic and thermodynamic factors. The lower energy barrier, exothermic nature, and greater INT stability of the d-to-l form conversion make it the favored pathway.

### Specialization of OpaE within the allantoin–hydantoin racemase subfamily

A BLAST search of OpaE in the UniProt database identified 497 homologous sequences, including characterized enzymes such as allantoin–hydantoin racemases (*Kp*AllR, *Pseudomonas fluorescens* allantoin racemase, *Aa*hydR, and *Sm*HydR) and NozR, which participates in the biosynthesis of the cyclo-d-Trp-d-Trp (6, 10, 11, 12, 13, 14). This distribution underscores an evolutionary link between OpaE and the hydantoin–allantoin racemase (HYD) subfamily, consistent with its UniProt classification. Previous evolutionary analyses suggest that the HYD subfamily likely diverged from the Asp/Glu family of enzymes to adapt to allantoin metabolism (6). The identification of OpaE and NozR reinforces the idea of functional diversification within the HYD subfamily, pointing to a broader substrate range and novel functional adaptations in this subfamily.

**Figure 7:**
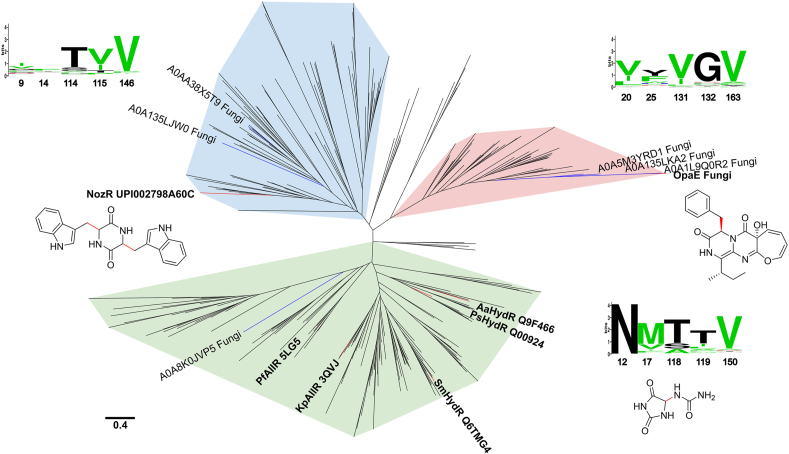
**Unrooted phylogenetic tree of the allantoin–hydantoin racemase subfamily from the Asp/Glu racemase superfamily.** Branches clustering with allantoin–hydantoin racemase, NozR, and OpaE are color-coded in *light green*, *blue*, and *red*, respectively. Adjacent sequence logos show the conservation of key residues involved in OpaE’s specific accommodation of hydrophobic substituents in HP1, HP2, and flexible residues in loop B. OpaE’s branch is labeled with its OpaE’s sequence numbering, NozR’s branch uses NozR’s corresponding numbering, and allantoin–hydantoin racemase’s branch is labeled with the *Kp*AllR numbering. Experimentally characterized enzymes are highlighted in *bold*, along with their PDB or UniProt accession numbers and corresponding substrates. The catalytic positions on the substrates are marked in *red*, with OpaE preferably catalyzing the conversion of d- to l-form, whereas other enzymes catalyze reversible reactions. Sequences from eukaryotic organisms, particularly fungi, are labeled for clarity.

To refine the analysis, the sequences were deduplicated, yielding 274 unique entries. A phylogenetic tree was then constructed, accompanied by a conservation analysis of key residues involved in OpaE's specific accommodation of hydrophobic substituents in HP1 (Ile20, Phe25), HP2 (Leu131, Phe132), and the flexible residues in loop B (Phe163). As shown in Figure 7, homologous sequences that cluster with OpaE, NozR, and allantoin–hydantoin racemases exhibit distinct amino acid characteristics. In OpaE-like enzymes, the HP1 and HP2 pockets predominantly consist of hydrophobic residues, including Val/Ile/Leu20 (89%), Tyr/Phe/Trp25 (74%), Val/Ile/Leu131 (95%), and Gly/Pro132 (86%), which contribute to substrate binding through hydrophobic interactions and structural flexibility. In contrast, allantoin–hydantoin racemases, such as *Kp*AllR, display different residues at equivalent positions: Asn/Asp12 (100%), Met17 (67.7%), Thr/Ser118 (84.2%), and Thr119 (55.6%). The crystal structure of *Kp*AllR reveals that Asn12, Thr118, and Thr119 form hydrogen bonds with the substrate (Fig. S3*B*), highlighting a structural distinction between these enzymes and OpaE. NozR-like enzymes exhibit residues more similar to those of OpaE, while retaining some features of allantoin–hydantoin racemases. The corresponding residues in this group are Ile/Val9 (44%), Phe/Leu14 (32%), Thr/Ser114 (79%), and Val/Ile115 (74%), reflecting this hybrid characteristic.

Interestingly, Phe163 on loop B shows a remarkable conservation as valine across most homologous sequences (84.7%). However, in all the fungal enzymes clustering with OpaE, this residue is substituted by phenylalanine or tyrosine, accompanied by a displacement of loops A and B (Fig. S8, *A* and *B*). These findings underscore the distinctiveness of fungal enzymes within this group, which aligns with the phylogenetic tree, where fungal enzymes form a relatively long branch separate from their bacterial counterparts. Previous studies have suggested that fungal Asp/Glu family enzymes were acquired through horizontal gene transfer from bacterial enzymes (15). The low number of fungal homologous sequences in our dataset (16 of 497) further supports this hypothesis, potentially explaining the unique features observed in fungal OpaE-like enzymes. These results suggest that OpaE represents a new branch of the Asp/Glu racemase superfamily.

## Discussion

The Asp/Glu racemase family enzymes are typically known for catalyzing bidirectional reactions because of their pseudosymmetrical structure and the minimal differences between substrate isomers, which enable both forms to bind effectively (16). In this study, we identify OpaE as a representative of fungal Asp/Glu racemases, catalyzing the “quasi-unidirectional” d-to-l form conversion of fungal NRP derivatives. While “quasi-unidirectional” catalysis has been observed in EcL-DER, where an “unbalanced” donor–acceptor pair (Thr83/Cys197) dictates specificity for l-form aspartic acid (17), OpaE represents a distinct case. Despite having a “balanced” donor–acceptor system (Cys90/Cys199), which would typically support bidirectional catalysis, OpaE still catalyzes the “quasi-unidirectional” d-to-l form conversion. Our docking and MD analyses, supported by mutagenesis, reveal that this quasi-unidirectional mode arises from the substrate’s binding mode, which disrupts the expected catalytic symmetry. Specifically, the d-form adopts a conformation that optimally facilitates proton transfer during epimerization. QM/MM analysis further shows that the d-to-l form conversion catalyzed by OpaE occurs with a significantly lower energy barrier, greater thermodynamic favorability, and enhanced INT stability compared with the l-to-d-form conversion. This finding unveils a novel mechanism for breaking catalytic symmetry within the Asp/Glu racemase family.

In NRP synthesis, epimerization typically occurs during the assembly process and is catalyzed by the E domain of NRPS (1, 2). In contrast, epimerization in ribosomal peptide synthesis typically happens after peptide assembly, as demonstrated by radical *S*-adenosylmethionine-dependent enzymes like PoyD14 and YydG, *α*/*β*-hydrolase family enzymes, such as BotH and LinH, and metallo-dependent peptide epimerases like MslH (18, 19, 20, 21, 22). These enzymes generally catalyze the conversion of l-amino acid residues to their d-form counterparts. Notably, OpaE also catalyzes epimerization after peptide assembly, similar to ribosomal peptide synthesis. However, unlike the mentioned enzymes, OpaE preferably converts the d-Phe moiety in 15-*epi*-oxepinamide E—produced by the NRPS enzyme OpaA—back to the l-configuration. It has been shown that the NRPS E domain is essential for NRP assembly, suggesting that the E domain in OpaA plays a similarly critical role. Meanwhile, 15-*epi*-oxepinamide E (d-form) exhibits very weak activity toward the methyltransferase OpaF, a tailoring enzyme acting after OpaE, thereby impeding the subsequent methylation step (3). This functional bottleneck likely provided selective pressure for the evolution of OpaE, which reverses the d-Phe moiety back to the l-configuration, restoring substrate compatibility for downstream processing. Our structural and computational analyses reveal the molecular basis for OpaE's substrate specificity and provide a paradigm for how fungi precisely design enzymes to control pathway flux and natural product biosynthesis. Notably, OpaE's ability to reverse amino acid stereochemistry (from d- to l-configuration) highlights its role in NRP biosynthesis as the first known member of the Asp/Glu racemase family to catalyze epimerization of NRP derivatives.

Asp/Glu racemase family enzymes typically act on relatively simple and small substrates, such as glutamine, aspartate, and allantoin. In contrast, OpaE catalyzes the epimerization of a structurally complex, NRPS-derived molecule, a fused tricyclic derivative with phenylalanine- and isoleucine-derived substituents. To accommodate this larger substrate, OpaE has evolved specialized HPs (HP1 and HP2) tailored for the phenylalanine and isoleucine side chains, along with conformational shifts in loop regions. These structural adaptations not only distinguish OpaE from other Asp/Glu racemase family members but also reflect its unique evolutionary trajectory, as evidenced by its longer branch length in phylogenetic analysis. Together, these distinctive catalytic properties position OpaE, and potentially its subgroup enzymes, as promising candidates for biocatalytic applications, particularly in selective asymmetric peptide epimerization.

## Experimental procedures

### Site-directed mutagenesis, protein expression, purification, and catalytic activity

Site-directed mutagenesis was carried out to introduce specific mutations into the OpaE gene using the QuikChange method. Specially designed primers (Table S3) with the desired mutations were used for PCR amplification of the plasmid containing the *opaE* sequence. The resulting mutated plasmids (Table S3) were then transformed into *Escherichia coli* BL21 (DE3) cells for gene expression. Protein production and purification were performed following methods described in our previous publications (3, 4). Expression was induced with 0.5 mM IPTG at 25 °C for 20 h. After expression, cells were harvested and lysed, and the proteins were purified using affinity chromatography. To achieve protein homogeneity and ensure optimal conditions for crystallization, the purified proteins were further processed using size-exclusion chromatography and concentrated in a buffer composition of 20 mM Tris (pH 9.0), 200 mM NaCl, and 5% glycerol. The catalytic activity of OpaE was based on the established protocols from our previous publications (3, 4). The reaction mixtures (50 μl) contained 50 mM Tris–HCl (pH 7.5), substrates at final concentrations from 0.5 to 2 mM, and purified enzymes at final concentrations from 1.3 to 11.5 μM. The incubations were carried out at 37 °C for 30 min. After the addition of 50 μl methanol and centrifugation, the supernatants were analyzed on HPLC. Substrate racemization was measured under optimized conditions to ensure accuracy.

### Crystallization, structure determination, and analysis

Crystallization trials were initially conducted using commercial crystallization kits to identify conditions that produced crystals. These conditions were optimized further to improve crystal quality. The final optimized crystallization conditions were 0.1 M sodium acetate (pH 4.6), 0.2 M ammonium sulfate, 15 to 20% (v/v) PEG 300, and 7.5% to 10% glycerol at 4 °C. Crystals were obtained using the hanging drop vapor diffusion method with a reservoir solution volume of 500 μl. Drops were prepared by mixing 2 μl of protein solution with 2 μl of reservoir solution.

X-ray diffraction data were collected at the P13 beamline of the Hamburg Synchrotron Radiation Facility (HASYLAB). The raw diffraction images were processed and scaled using XDSapp3 software to determine the unit cell parameters and to convert the images into a set of intensities (23). The initial structural model was generated through molecular replacement with the Phaser program, utilizing structural predictions from AlphaFold2 as a reference (24, 25). Subsequent structure refinement was carried out using the Phenix suite, which iteratively adjusted the model to improve the fit between the observed and calculated diffraction data (26). Finally, structural analysis and visualization of the refined model were performed using PyMOL to interpret the atomic arrangement and gain insights into the protein's three-dimensional structure. Data collection and structure refinement statistics are summarized in Table S1.

### Molecular docking and MD simulations

Preparation of the protein and ligand was completed using AutoDock Tools (ADT 4.2) to generate pdbqt files and assign Gasteiger charges (27). AutoGrid was used to create the grid map, with the grid box set to dimensions of 60 × 60 × 60 *xyz* points, a grid spacing of 0.375 Å, and centered at coordinates (−15.094, 53.091, 55.105). Docking was performed with AutoDock using the genetic algorithm for optimization, treating both protein and ligand as rigid. Poses were evaluated based on binding energy and their alignment with catalytic amino acids. The pose with the most favorable binding energy and optimal positioning relative to catalytic residues was selected for further analysis.

MD simulations of the complex were carried out using the GPU-accelerated Amber22 package (28), based on the docking results. The protonation states of charged amino acid residues (Asp, Glu, and His) were determined using PROPKA at pH 7.0 (29) and further refined based on their hydrogen-bonding networks. For the catalytic residues Cys90 and Cys199, their protonation states were assigned according to their roles in catalysis, either as proton acceptors or donors. The force field parameters were applied using Amber ff14SB (30) for the protein and GAFF (31) for the substrate, with RESP charges (32) for the substrate calculated at the B3LYP/6-31G∗ level of theory. The protein–substrate system was then solvated in a 16 Å TIP3P water box (33), and Na^+^ ions were added to neutralize the system's overall charge. After system preparation, energy minimization was performed using a combination of steepest descent and conjugate gradient methods to relax the structure. The system was then heated to 300 K in the NVT ensemble, followed by equilibration in the NPT ensemble with a weak restraint of 25 kcal/mol/Å^2^ applied to the protein. Finally, 100 ns of productive MD simulations were conducted in the NPT ensemble without any harmonic restraints. Long-range electrostatics were handled using the particle mesh Ewald method (34), covalent bonds involving hydrogen were constrained with the SHAKE algorithm (35), and a 2 fs time step was used throughout the simulations. Trajectory analyses were performed using standard tools in Amber and VMD (36). The protein backbone RMSD fluctuations during the MD simulations are presented in Fig. S7.

### QM/MM and metadynamics simulations

QM/MM simulations were conducted using the CP2K 6.0 package, which integrates the QM program QUICKSTEP with the MM driver FIST (37). Representative snapshots from the MD-equilibrated systems were used as starting points. The QM region, which includes the catalytic residues (Cys90, Cys199) and the substrate, was treated using density functional theory at the BLYP level with a dual Gaussian and plane-wave basis set. A Gaussian triple-ζ valence polarized (TZV2P) basis set was used to expand the wave function, whereas an auxiliary plane-wave basis set with a density cutoff of 360 Ry and the GTH pseudopotential was used to converge the electron density (38). The remaining MM region was treated classically using the same parameters as in the MD simulations. The system was placed within a 20.0 × 20.0 × 20.0 Å^3^ supercell, with electrostatic coupling between the QM and MM regions handled *via* a real-space multigrid technique (39). Simulations were performed under the NVT ensemble for 10 ps with a 0.5 fs time step.

To investigate the molecular mechanism and free energy profile, QM/MM metadynamics simulations (40) were conducted using the PLUMED2 plugin (41). The first and second reaction steps were driven by collective variables (CV1 and CV2), where CV1 tracked the distance difference between substrate H15 to C15 and to the catalytic cysteine SG, and CV2 monitored the distance difference between cysteine HG to SG and substrate C15. Both CVs were set with a Gaussian width of 0.15 Å, a deposition time of 12.5 fs, and a hill height of 0.6 kcal/mol. The free energy barrier was determined by tracing the minimum free energy path (42), with convergence achieved upon crossing the transition state.

### Phylogenetic analysis

Homologous sequences were identified by performing a BLAST search against the UniProt database (5) using the OpaE sequence, with an E-value threshold of less than 10^−5^. These sequences were then clustered using CD-HIT, applying a 0.7 similarity threshold, and only sequences with lengths between 200 and 400 amino acids were retained (43). Sequence alignments were performed using MAFFT for both fast and local alignment strategies, and Jalview was used for subsequent analysis of the alignments (44, 45). Evolutionary relationships were assessed using IQ-TREE, and the best-fit molecular evolution model was selected through ModelFinder Plus (46). The resulting phylogenetic tree was visualized with FigTree (47).

## Data availability

Coordinates and structure factors for OpaE have been deposited to the PDB (www.rcsb.org), under the accession code 9QBX.

## Supporting information

This article contains supporting information.

## Conflict of interest

The authors declare that they have no conflicts of interest with the contents of this article.
