# Supplementary material for: Molecular insights into the substrate recognition and quasi-unidirectional catalysis of OpaE, an atypical fungal enzyme from the Asp/Glu racemase family
Source: J Biol Chem. 2025 Oct 16;301(12):110828. doi: 10.1016/j.jbc.2025.110828 (PMC12661438; doi:10.1016/j.jbc.2025.110828)
Supplement: Supporting Tables and Figures [file mmc1.pdf]

Supplementary Information

Molecular Insights into the Substrate Recognition and 'quasi-unidirectional' Catalysis of OpaE, an Atypical Fungal Enzyme from the Asp/Glu Racemase Family

Xuehua Zheng[a,b,#], Zhiyong Guo[c, #], Leyao Chen[d,#], Haowen Wang[d], Johannes Freitag[a], Aitao Li[c], Gert Bange[a,e,*], Shu-Ming Li[d,*], Liujuan Zheng[a,e,*]

[a]*Marburg University, Center for Synthetic Microbiology (SYNMIKRO) & Department of Chemistry, Karl-von-Frisch Strasse 14, 35043 Marburg, Germany*
[b]*Guangzhou Municipal and Guangdong Provincial Key Laboratory of Molecular Target & Clinical Pharmacology, the NMPA and State Key Laboratory of Respiratory Disease, School of Pharmaceutical Sciences, Guangzhou Medical University, Guangzhou 511436, China*
[c]*State Key Laboratory of Biocatalysis and Enzyme Engineering, Hubei Key Laboratory of Industrial Biotechnology, School of Life Sciences, Hubei University, Wuhan 430062, China*
[d]*Marburg University, Department of Pharmacy, Institute of Pharmaceutical Biology and Biotechnolohy, Robert-Koch Straße 4, 35037 Marburg, Germany*
[e]*Max-Planck Institute for Terrestrial Microbiology, Molecular Physiology of Microbes, Karl-von-Frisch Strasse 14, 35043 Marburg, Germany*

[#] These authors contribute equally to this work.
[*] Corresponding author: gert.bange@synmikro.uni-marburg.de, shuming.li@staff.uni-marburg.de, Liujuan.Zheng@mpi-marburg.mpg.de

Table S1. Data Collection and Refinement Statistics

**Data collection**	
Wavelength (Å)	0.97626
Resolution (Å)	49.1 - 2.65 (2.81 - 2.65)
Space group	$P3_221$
a, *b*, *c* (Å)	118.87, 118.87, 213.58
α, β, γ (°)	90, 90, 120
Total reflections	1037747 (170854)
Unique reflections	97817 (15819)
Multiplicity	10.7 (11.0)
Completeness (%)	100 (100)
Mean I/sigma(I)	21.57 (2.83)
R_{merge}	0.079 (0.783)
$CC_{1/2}$	0.999 (0.825)
**Refinement**	
R_{work}/R_{free}	0.2302/0.2492
No. of atoms	3855
macromolecules	3840
ligands	15
solvent	0
R.m.s. deviations	
Bond lengths (Å)	0.008
Bond angles (°)	1.00
Ramachandran favored (%)	95.00
Rotamer outliers (%)	0.20
Clash score	7.08
B factors (Å^2)	69.92

Table S2. Dali Analysis of Structural Similarity between OpaE and Related Enzymes

No.	PDB ID	Z-score	RMSD (Å)	Identity (%)	Aligned Residues	Description
1	2EQ5	22.8	2.1	21	206	Unreported
2	3QVL	22.1	2.6	18	222	Allantoin racemase *Kp*AllR
3	5LG5	21.9	2.9	22	217	Allantoin racemase *Pf*AllR
4	3EIS	15.6	3.0	11	196	Arylmalonate decarboxylase
5	2XED	15.6	2.9	13	201	Maleate Isomerase
6	5ELM	15.5	2.8	11	196	Aspartate racemase EcL-DER
7	4IX1	15.3	3.2	11	195	Unreported
8	4FQ5	15.3	3.0	16	199	Maleate isomerase
9	3S81	15.3	3.1	17	197	Unreported
10	2ZSK	15.2	2.9	12	197	Aspartate racemase
11	2DGD	14.8	3.2	12	198	Unreported
12	1JFL	14.3	2.8	15	191	Aspartate racemase
13	2JFU	14.3	3.4	12	201	Glutamate racemase
14	5B19	14.2	3.1	13	193	Aspartate racemase
15	5IJW	13.6	4.0	11	206	Glutamate racemase
16	2DWU	13.6	3.7	11	202	Glutamate racemase
17	5W16	13.3	3.8	11	200	Unreported
18	5WXY	13.2	3.1	10	192	Aspartate racemase
19	3OUT	13.2	4.1	12	202	Unreported
20	2JFQ	13.2	4.0	12	200	Glutamate racemase
21	7UJ5	11.8	4.0	14	193	Unreported
22	1B74	11.4	3.8	11	185	Glutamate racemase
23	2JFN	11.4	4.3	12	193	Glutamate racemase

Table S3. Plasmids and Primers Used in This Study

Plasmids	Description	Primers[a]
pLZ304	pET-24d-Nhis-OpaE, a 825 bp fragment of *opaE* from cDNA of *A. ustus* 3.3904, with BsaI inserted in pET-24d	CCGCAAGCTTGTCGACGGAGCTCGAATTCCTAGA TTTCAATCCTTCCAC
		TGGTGGACAGCAAATGGGTCGCGGATCCATGGGT CCTCTCCGTGTTTT C
pLZ132	Mutation of C90S in pLZ304	CTTATCATCAACAGTTTTGGCGAT
		ATCGCCAAAACTGTTGATGATAAG
pLZ133	Mutation of C199S in pLZ304	GTTCTTGTCCTGGGCAGTACG
		CGTACTGCCCAGGACAAGAAC
pLZ134	Mutation of C90S/C199S in pLZ304	CTTATCATCAACAGTTTTGGCGAT[b]
		ATCGCCAAAACTGTTGATGATAAG[b]
pLZ 244	Mutation of F163V in pLZ304	ATGGCGGTTAACGATGATCTTACGCCCATGGA
		ATCGTTAACCGCCATATCGACAACTGGATGCGC
pLZ247	Mutation of F163Y in pLZ304	ATGGCGTACAACGATGATCTTACGCCCATGGA
		ATCGTTGTACGCCATATCGACAACTGGATGCGC

[a]Primer sequences are provided in the 5'→3' direction, with forward primers (F) in the upper row and reverse primers (R) in the lower row for each pair.

[b]using pLZ133 as the template for PCR.

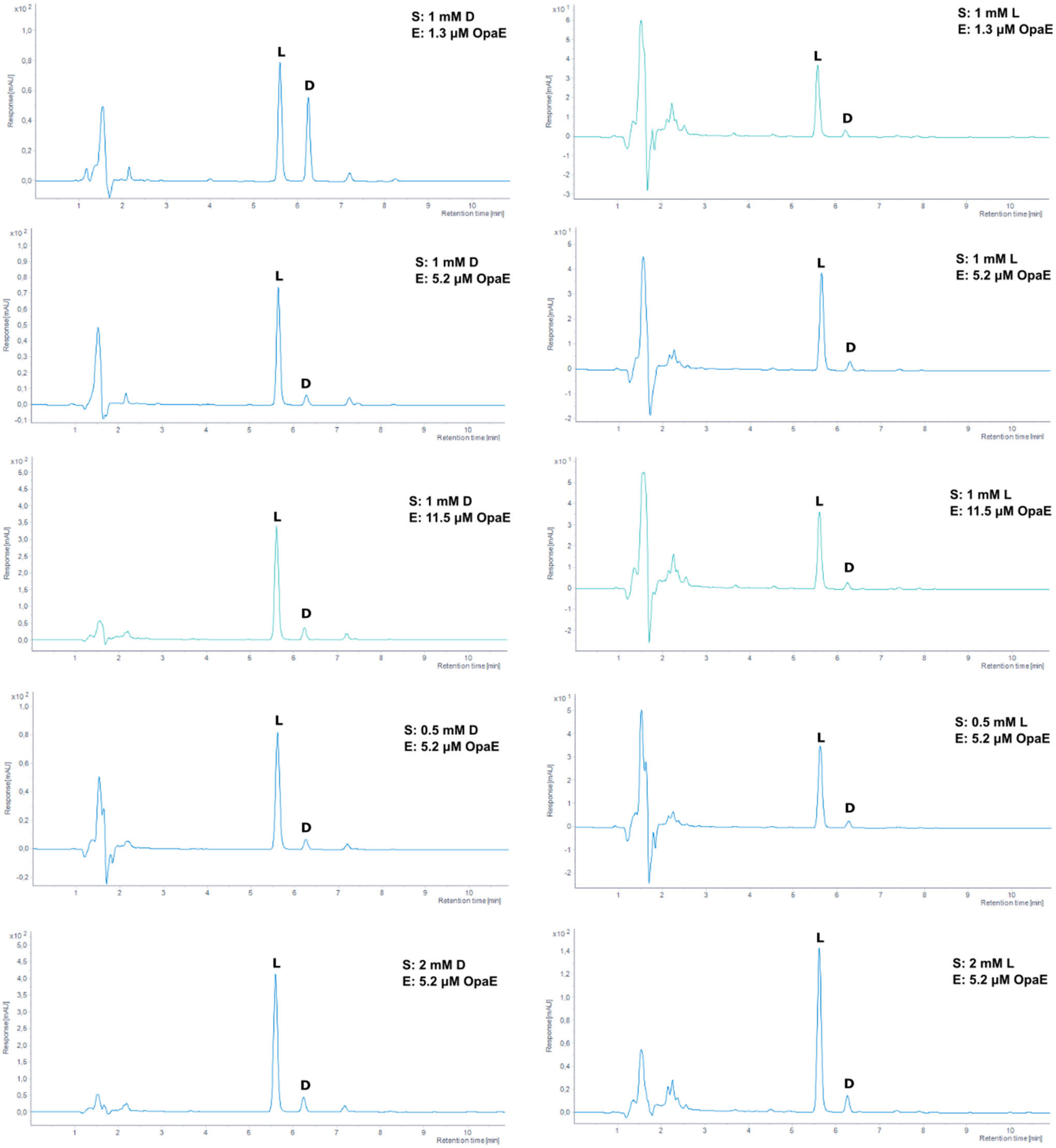

Figure S1. HPLC Profiles of D-to- L and L-to-D Conversions at Varying Substrate and Enzyme Concentrations D and L refer to 15-*epi*-oxepinamide E and oxepinamide E, respectively. Substrate (S) and enzyme (E) concentrations used in each reaction are indicated in the top right corner of the corresponding chromatograms.

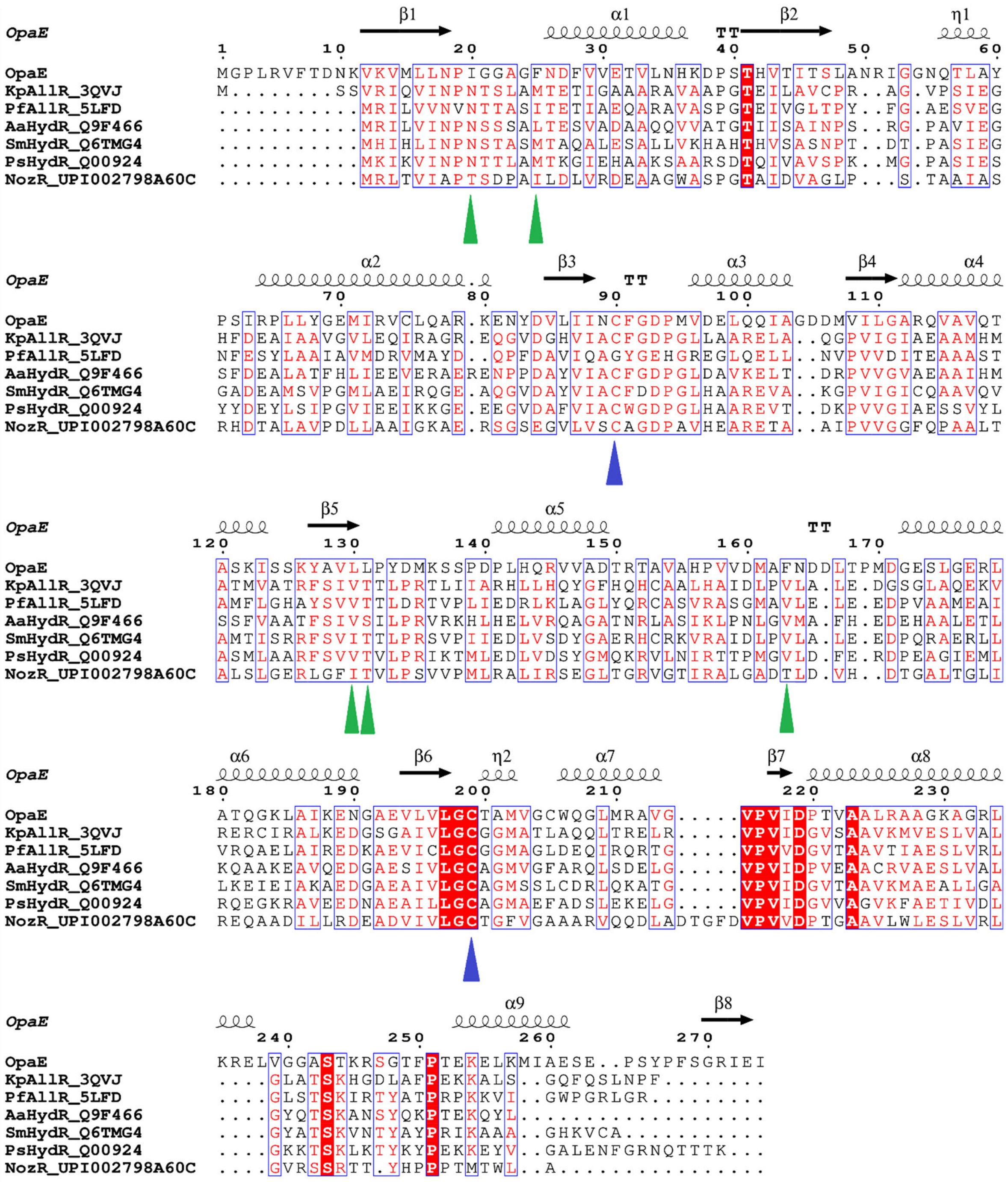

Figure S2. Sequence Alignment of OpaE with Selected Members of the Asp/Glu Racemase Superfamily

The sequences were aligned using MAFFT, and the alignment figure was generated using the ESPript server. The analyzed sequences include OpaE and related experimentally characterized proteins, labeled with their respective PDB or UniProt accession numbers. Completely conserved residues are highlighted with red boxes containing white lettering, while regions of sequence similarity are indicated by red lettering on a white background. Blue triangles mark the two conserved cysteine residues, while green highlights denote key residues involved in OpaE's specific accommodation of hydrophobic substituents in HP1 and HP2, and the flexible residue in loop B. The numbering and secondary structural elements correspond to those in the OpaE structure presented in this study.

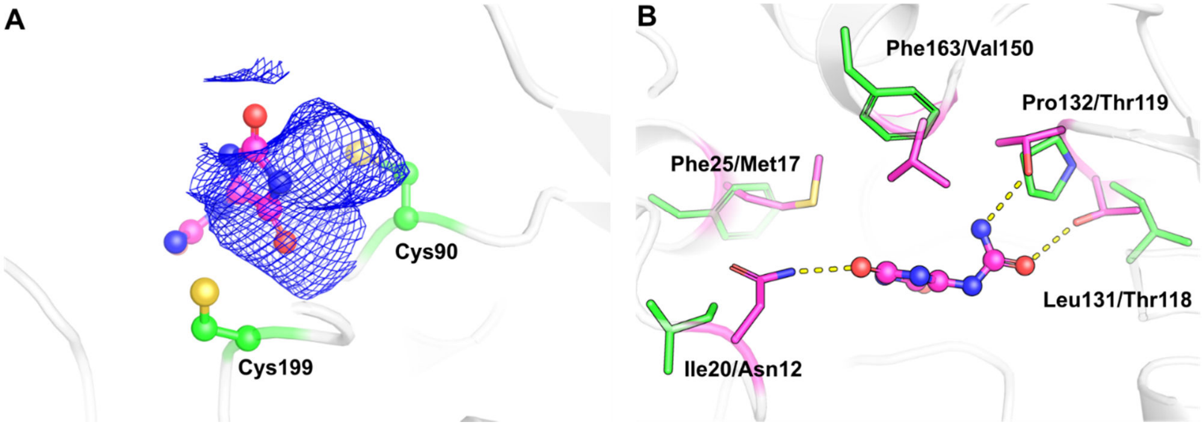

Figure S3. Structural Overlay of OpaE with Substrate-bound *Kp*AllR

(A) Electron density (mFo-DFc at 2.0 σ) observed between Cys90 and Cys199 in OpaE. Although low resolution hindered accurate fitting of potential molecular models, this density suggests a possible substrate binding site, as indicated by the overlay with *Kp*AllR (PDB ID: 3QVK), where the substrate (magenta) occupies a corresponding position.

(B) The OpaE binding pocket is enriched in hydrophobic residues. Key active site residues in OpaE (green) are compared with those in *Kp*AllR (magenta), with the substrate (magenta) bound to *Kp*AllR shown for reference.

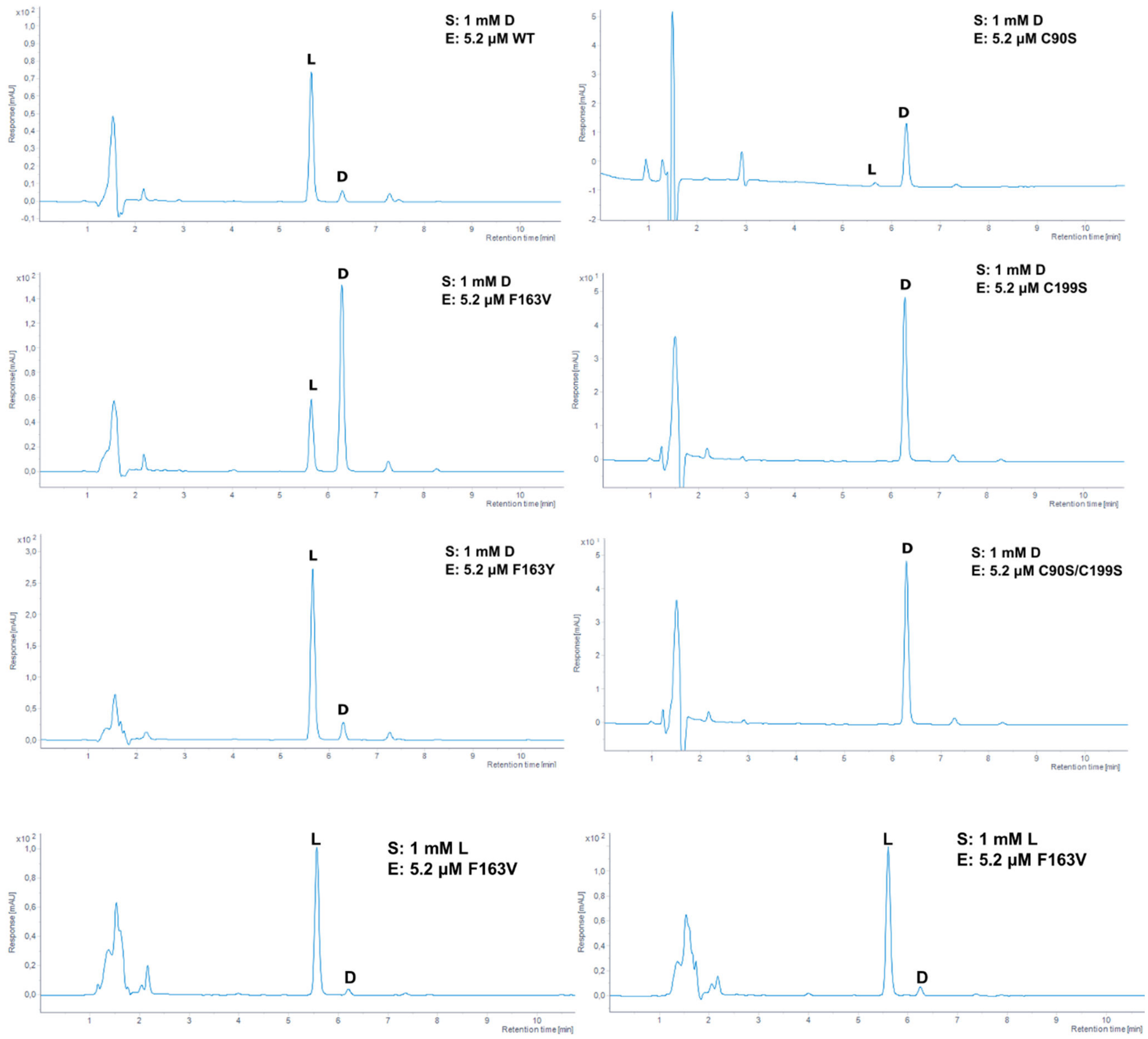

Figure S4. HPLC Profiles of OpaE Mutants Targeting Catalytic Cysteines and Loop B Residue F163

D and L refer to 15-*epi*-oxepinamide E and oxepinamide E, respectively. Substrate (S) and enzyme (E) concentrations used in each reaction are indicated in the top right corner of the corresponding chromatograms.

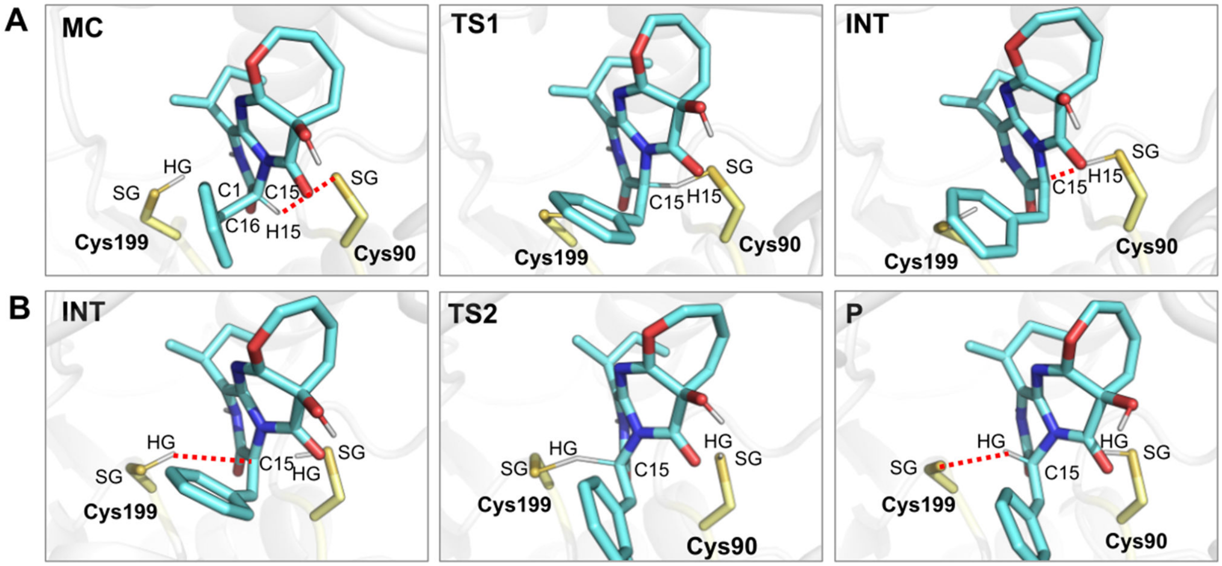

Figure S5. Representative Active Site Structures of L- to D- Conversion Catalyzed by OpaE

(A) Active site structures along the first step of the catalytic pathway, showing the Michaelis complex (MC), the transition state of the first reaction step (TS1), and the reaction intermediate (INT).

(B) Active site structures along the second step of the catalytic pathway, showing the reaction intermediate (INT), the transition state of the second reaction step (TS2), and the final product (P).

For clarity, hydrogen atoms attached to carbon atoms have been omitted. Red dashed lines indicate bond formation and cleavage events between the catalytic Cys residue and the substrate.

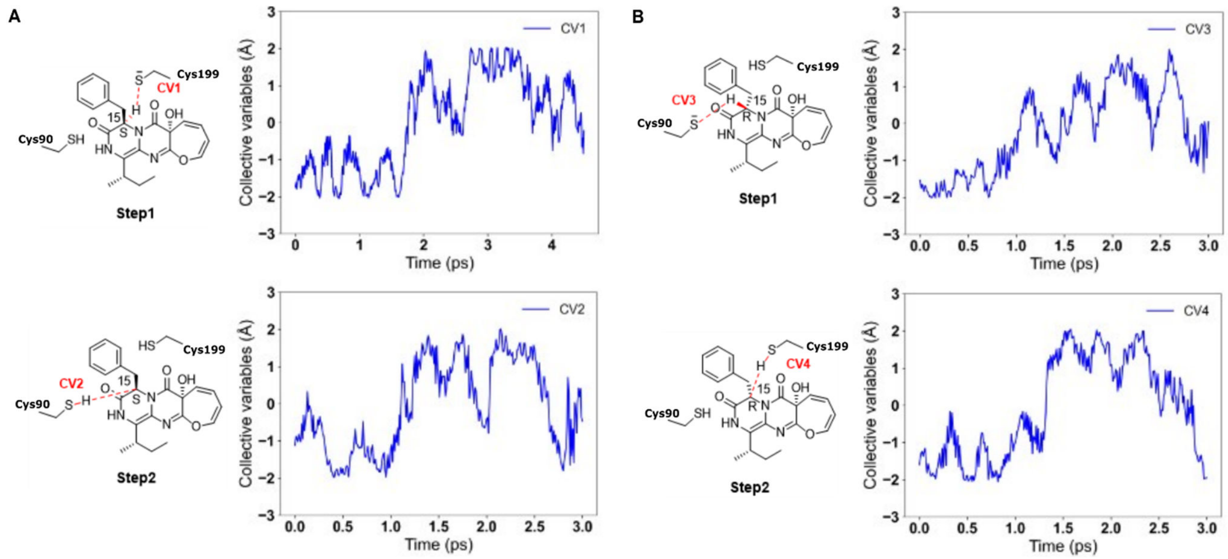

Figure S6. Collective Variables Monitoring Proton Transfers during QM/MM Metadynamics Simulation

(A) Schematic representation of catalytically relevant distances and collective variables (CVs) during the D- to L-conversion (left panel) and their evolution over time (right panel). CV1 represents the difference between the distances of the substrate H15 to its C15 and to the SG of Cys199 in step 1, while CV2 represents the difference between the distances of the Cys90 HG to its SG and to the substrate C15 in step 2.

(B) Schematic representation of catalytically relevant distances and collective variables (CVs) during the L- to D-conversion (left panel) and their evolution over time (right panel). CV3 corresponds to the difference between the distances of its H15 to C15 and to SG of Cys90 in step 1, while CV4 represents the difference between the distances of Cys199 SG to its SG and to the substrate C15 in step 2.

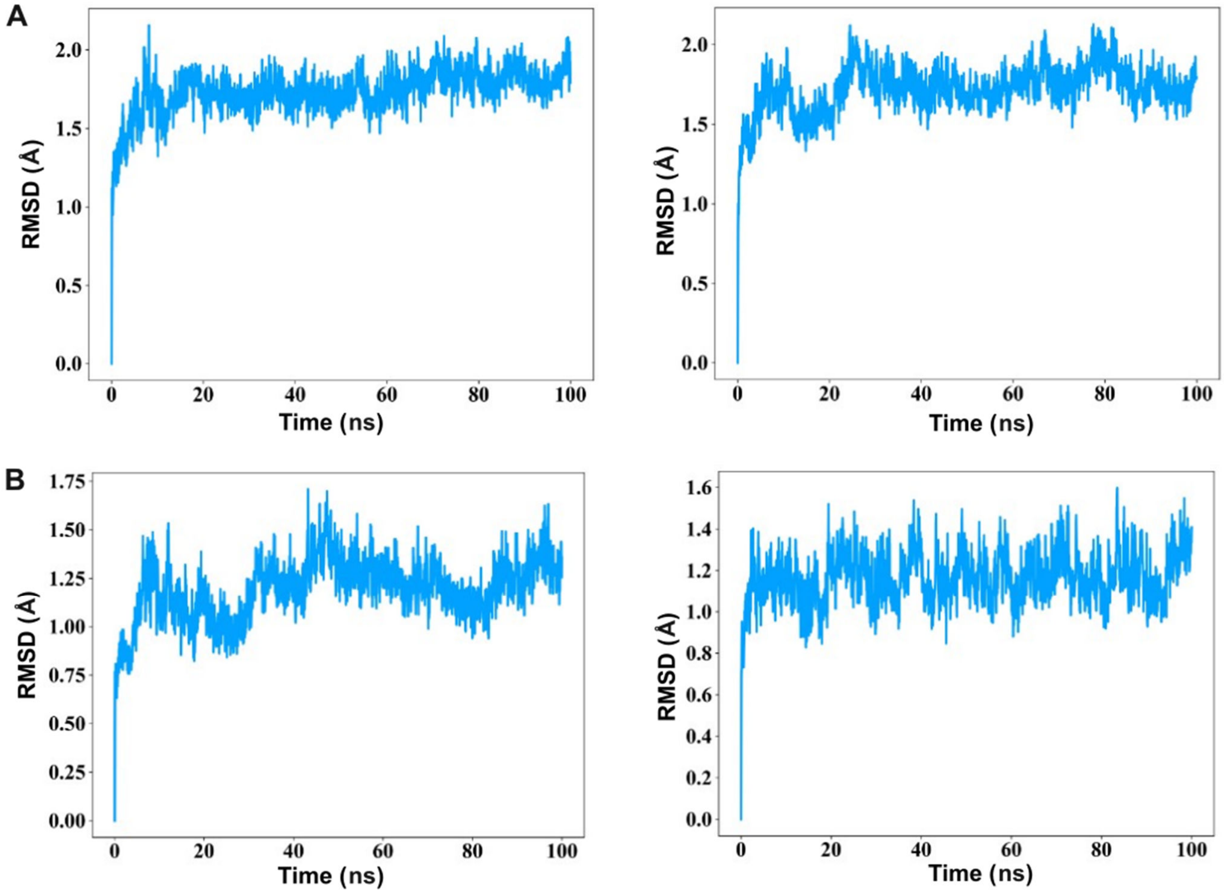

Figure S7. Protein Backbone RMSD Fluctuations in MD Simulations

(A) MD simulation of OpaE/D-form (left panel) and OpaE/L- form (right panel) complex derived from docking simulations

(B) MD simulation of intermediate state (derived from QM/MM simulation) during D- to L- (left panel) and L- to D- (right panel) conversion in OpaE catalysis.

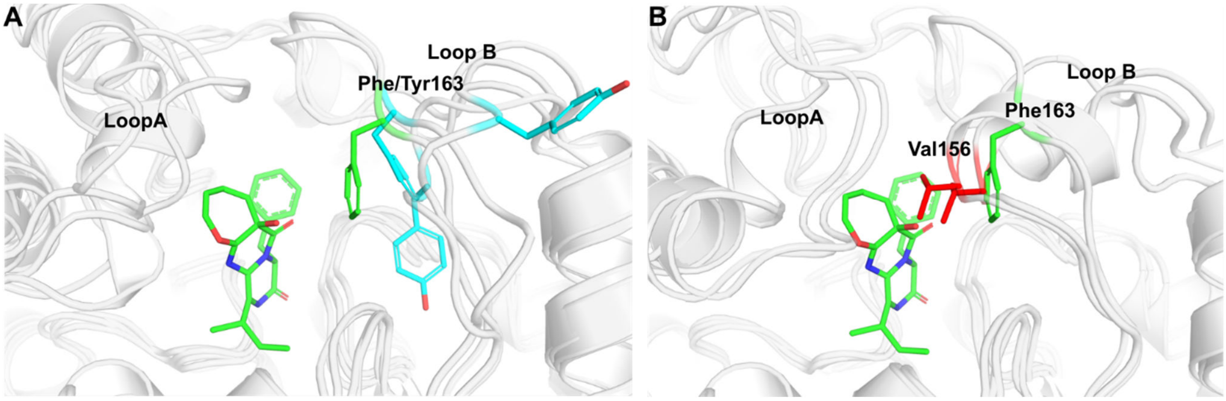

Figure S8. Structural Comparison of OpaE with Its Closely Related Enzymes

(A) Structural analysis of fungal enzymes clustered with OpaE (AlphaFold-predicted structures; UniProt IDs: A0A135LKA2, A0A1L9Q0R2, A0A5M3YRD1) shows phenylalanine or tyrosine (cyan) at the position corresponding to Phe163 (green) in loop B. These structural features are accompanied by notable displacements in loops A and B, potentially influencing substrate binding.

(B) AlphaFold-predicted structures bacterial enzymes clustered with OpaE (UniProt IDs: A0A5C7BE58, A0A7X0MUQ5) display valine at the position corresponding to Phe163 (green) in loop B, along with distinct loop A and B conformations when compared to fungal enzymes.

The substrate (green) bound to OpaE is used as a positional reference to highlight differences between fungal and bacterial enzyme structures and their associated loop dynamics.

Cartesian Coordinates of Key Reaction State Structures from QM/MM-Based Metadynamics Simulations

Conversions from D- to L- Form

MC:

S	6.215847	9.955334	6.773678
S	13.634920	9.250170	10.659099
O	6.795310	9.461712	14.647121
O	10.618902	8.464835	8.263532
O	10.481712	11.455912	12.022600
O	9.564095	10.490322	14.727536
N	8.959380	7.604360	9.522679
N	9.317561	9.666491	11.275938
N	7.542782	8.791705	12.624697
C	6.510591	9.719025	4.974141
C	13.971650	7.536699	9.921884
C	9.875734	8.643563	9.193074
C	8.120546	7.688038	10.618420
C	8.267650	8.739124	11.487790
C	9.774245	9.966850	9.896925
C	7.746990	9.638775	13.529001
C	8.768212	10.664160	13.527568
C	9.618413	10.612054	12.219810
C	5.829453	10.393434	14.962323
C	5.873736	11.743018	14.773190
C	6.901082	12.446927	14.105947
C	8.094850	12.045336	13.629628
C	9.072995	10.946311	8.952777
C	9.202988	12.475664	9.138182
C	10.374093	13.055724	8.637618
C	10.540231	14.436610	8.557239

C	9.489323	15.294241	8.933214
C	8.301201	14.762138	9.410257
C	8.178138	13.349584	9.552142
C	7.180047	6.515706	10.901257
C	7.735803	5.071717	10.800546
C	9.039088	4.746939	11.597129
C	6.015050	6.789943	9.872966
H	6.354993	10.631075	4.499026
H	5.738194	8.981300	4.686117
H	6.674587	8.714104	7.072845
H	13.266981	6.848185	10.381991
H	15.028208	7.186480	10.000420
H	8.867038	6.800540	8.875692
H	10.894652	10.269396	9.986560
H	4.971792	9.954477	15.500973
H	5.009249	12.263118	15.077868
H	6.653377	13.502400	14.033012
H	8.693308	12.774717	13.051270
H	7.998824	10.743479	8.692450
H	9.605688	10.677484	8.022353
H	11.119320	12.347248	8.281315
H	11.444245	14.861453	8.194773
H	9.561099	16.359049	8.792525
H	7.432137	15.442339	9.631247
H	7.238138	12.909396	9.773383
H	6.822594	6.677788	11.930058
H	6.947171	4.339466	10.997252
H	7.958287	4.838744	9.782363
H	9.180707	3.640950	11.784581

H	9.848228	5.166391	10.971323
H	9.059686	5.173733	12.584295
H	10.149753	11.306497	14.755998
H	5.607715	7.743013	10.194648
H	6.371325	6.969421	8.862867
H	5.267079	5.971622	9.855577
H	7.577613	9.452206	4.742262
H	13.786406	7.320467	8.782838

TS1:

S	6.861562	9.883380	6.971136
S	13.530587	10.110232	10.164664
O	7.330159	8.572312	14.964884
O	10.421205	9.366678	8.052476
O	10.683337	11.278123	12.712779
O	10.176867	9.342660	15.063872
N	9.482719	7.972419	9.454033
N	9.574450	9.695894	11.562201
N	8.137482	8.170811	12.783692
C	7.170258	10.318848	5.102269
C	13.946456	8.361132	9.943554
C	10.069679	9.158893	9.188468
C	8.795341	7.567041	10.572769
C	8.868418	8.465981	11.663691
C	10.353251	10.076324	10.340914
C	8.228424	8.862391	13.885052
C	9.236023	9.903141	14.102136
C	9.860278	10.370347	12.711303
C	6.229807	9.424017	15.035591

C	6.341314	10.778278	15.042277
C	7.556858	11.551866	15.078719
C	8.788242	11.174000	14.757519
C	10.421394	11.561996	9.897692
C	9.334430	12.572586	10.031712
C	9.626741	13.798062	9.426864
C	8.945423	14.998344	9.674988
C	7.841549	15.008395	10.508657
C	7.445442	13.748814	10.982303
C	8.204258	12.637496	10.820857
C	8.153964	6.268331	10.690127
C	8.970866	5.044939	10.292505
C	10.298104	5.000297	11.007182
C	6.740956	6.184683	10.195915
H	6.930236	11.339191	5.019141
H	6.426731	9.588335	4.764089
H	7.984468	10.512548	7.379570
H	13.009768	7.706025	10.203711
H	14.742522	7.948915	10.549564
H	9.302126	7.395872	8.646575
H	11.743947	9.814591	10.539282
H	5.257477	8.895049	14.919828
H	5.446176	11.395976	15.134737
H	7.446467	12.658069	15.248246
H	9.578486	11.924047	14.923539
H	10.579168	11.596614	8.729216
H	11.339073	11.903295	10.419389
H	10.483305	13.856523	8.737400
H	9.285425	15.919601	9.173491

H	7.229578	15.906260	10.753501
H	6.518090	13.618091	11.561804
H	7.730750	11.739890	11.263800
H	8.022953	6.220204	11.807651
H	8.400539	4.146639	10.620577
H	9.219882	4.912197	9.257506
H	10.862140	4.080398	10.801943
H	10.925169	5.871596	10.666567
H	10.113741	5.112255	12.046217
H	10.865314	10.034305	15.103593
H	6.401926	5.275550	10.623769
H	6.094509	7.061425	10.482123
H	6.625991	6.076832	9.077434
H	8.212795	9.990104	4.848272
H	14.136210	8.022809	8.850300

INT:

S	8.521205	9.885038	13.051309
S	6.330375	10.121853	6.024876
O	13.597732	9.833084	8.437643
O	6.333087	9.611500	10.230874
O	9.714196	12.331999	7.242337
O	12.544442	12.091337	7.179681
N	8.115229	8.324609	9.594746
N	9.483170	10.509520	8.585742
N	11.481970	9.119131	8.748031
C	6.981056	10.719518	13.805021
C	5.641967	8.403437	6.047037
C	7.462725	9.604698	9.680392

C	9.453979	8.212939	9.379108
C	10.140710	9.289055	8.901479
C	8.123520	10.653097	9.042541
C	12.250085	10.116136	8.446079
C	11.741768	11.476566	8.278047
C	10.219088	11.428899	7.918669
C	14.329040	10.161537	9.579917
C	13.984896	11.081495	10.501415
C	12.899467	12.012050	10.435578
C	11.911901	12.205406	9.544065
C	7.403004	11.985229	8.955675
C	7.761178	13.217085	9.815288
C	6.964893	14.372826	9.618308
C	7.377492	15.564487	10.207777
C	8.423625	15.560423	11.105245
C	9.112891	14.363634	11.400421
C	8.772782	13.177750	10.741986
C	10.027245	6.820527	9.505295
C	9.101020	5.649653	9.264889
C	9.879453	4.327162	9.049376
C	10.628131	6.640882	10.880190
H	6.986499	11.682056	13.325869
H	7.257176	10.627807	14.868043
H	8.348253	10.418325	11.644456
H	6.441817	7.613179	5.887165
H	5.131079	8.419494	5.131957
H	7.092019	10.187257	7.217363
H	7.765983	7.696528	10.354732
H	15.326867	9.633209	9.596262

H	14.573997	11.091919	11.423891
H	12.766827	12.549531	11.353251
H	11.171202	12.946175	9.790351
H	6.363657	11.711287	9.350131
H	7.554558	12.326760	7.914444
H	6.049067	14.296965	8.978983
H	6.825200	16.499119	10.204402
H	8.638386	16.412891	11.695016
H	9.815607	14.308106	12.220284
H	9.368790	12.238921	10.894269
H	10.829865	6.718090	8.760509
H	8.465936	5.583205	10.144587
H	8.495900	5.803521	8.329689
H	9.318336	3.500880	8.574988
H	10.774274	4.422050	8.385912
H	10.235130	4.007578	10.033134
H	12.726290	13.070092	7.314538
H	9.900083	6.696018	11.691319
H	11.109412	5.684753	11.027946
H	11.343986	7.417650	11.051777
H	6.033074	10.226991	13.640308
H	4.939913	8.057065	6.829059

TS2:

S	8.200706	9.636375	13.687728
S	6.590759	9.334441	6.824335
O	13.603195	9.826119	7.751442
O	6.440652	9.635937	10.286799
O	10.124598	12.690611	8.003457

O	12.810755	12.227159	8.055854
N	8.312730	8.332182	9.930315
N	9.683676	10.572553	8.870482
N	11.498304	9.055864	8.295459
C	6.728559	10.593832	13.938903
C	5.722250	7.945155	6.053409
C	7.709691	9.572616	10.002964
C	9.510301	8.157743	9.286359
C	10.225363	9.236196	8.769066
C	8.372694	10.668264	9.354487
C	12.314431	10.014495	8.266977
C	11.979749	11.335876	8.781269
C	10.512953	11.633715	8.479344
C	14.637180	10.174879	8.637885
C	14.566885	10.625174	9.933597
C	13.454367	11.136424	10.756573
C	12.262415	11.498932	10.224176
C	7.538334	11.787611	8.823641
C	7.608720	13.132946	9.541945
C	7.162754	14.349340	8.983982
C	7.588612	15.585484	9.525141
C	8.321885	15.542987	10.726510
C	8.615932	14.306224	11.400654
C	8.281164	13.106230	10.784291
C	10.090714	6.781969	9.322824
C	9.097178	5.639209	9.183575
C	9.783769	4.330847	8.844098
C	11.037889	6.656149	10.634074
H	6.741939	11.626879	13.501196

H	6.430394	10.721412	14.965159
H	8.527947	10.708083	11.524040
H	6.342127	7.025774	6.064448
H	5.628237	8.284628	5.034840
H	6.521976	8.997577	8.131789
H	7.831667	7.543243	10.316202
H	15.589006	9.883200	8.175845
H	15.579052	10.720371	10.377059
H	13.691729	11.403937	11.831861
H	11.460884	11.747068	10.840688
H	6.549736	11.362086	8.761049
H	7.868049	11.981552	7.778325
H	6.542939	14.364191	8.065403
H	7.217647	16.526127	9.108656
H	8.665695	16.456770	11.257794
H	9.209169	14.313865	12.312029
H	8.539609	12.168540	11.239230
H	10.724936	6.690969	8.441535
H	8.488236	5.527924	10.098638
H	8.393336	5.776165	8.328609
H	9.216304	3.473872	9.271558
H	9.893104	4.255798	7.699141
H	10.808972	4.183911	9.305872
H	12.282213	13.003753	7.773281
H	10.431901	6.657644	11.591189
H	11.582679	5.724852	10.541986
H	11.728975	7.476818	10.685519
H	5.786961	10.198833	13.418237
H	4.772064	7.662689	6.601095

P:

S	8.038135	9.813562	12.960869
S	6.054673	10.065500	6.089211
O	13.623176	9.855960	8.488616
O	6.234031	9.455185	9.439443
O	9.999289	12.649004	7.958548
O	12.644083	12.339460	7.916893
N	8.071405	8.234184	8.995402
N	9.606812	10.503857	8.793950
N	11.552602	9.033033	8.893417
C	6.629351	10.528921	13.880474
C	5.645669	8.276528	6.102901
C	7.424356	9.448033	9.119514
C	9.419126	8.135048	9.167105
C	10.194857	9.255579	9.007898
C	8.317605	10.646290	9.410242
C	12.312327	10.067774	8.731905
C	11.892476	11.550999	8.811322
C	10.412290	11.589240	8.427396
C	14.628054	10.425461	9.297746
C	14.538893	11.235438	10.391949
C	13.275188	11.755621	10.935775
C	12.092305	11.874211	10.281116
C	7.433600	11.902198	9.143780
C	7.806193	13.151015	9.814065
C	7.275214	14.368851	9.249311
C	7.405481	15.567913	9.943766
C	8.065123	15.620896	11.220748

C	8.614660	14.451884	11.721933
C	8.541877	13.246376	11.028244
C	10.019534	6.775635	9.373935
C	8.996772	5.639689	9.484239
C	9.716290	4.379317	9.933263
C	10.932066	6.718981	10.590271
H	6.707858	11.642315	13.873595
H	6.542826	10.239655	14.931321
H	8.546081	10.488352	10.483911
H	6.404559	7.643018	6.537750
H	5.425627	7.906133	5.068679
H	5.638915	10.454755	7.294728
H	7.603277	7.322187	9.059887
H	15.560039	10.234891	8.743949
H	15.472979	11.468162	10.911294
H	13.404324	12.139238	12.014089
H	11.235680	12.203741	10.810750
H	6.470798	11.550339	9.519991
H	7.318433	11.945848	8.010616
H	6.706714	14.325331	8.293673
H	6.935292	16.485744	9.688303
H	8.107014	16.575064	11.814829
H	9.142560	14.395666	12.656617
H	9.045777	12.348089	11.384467
H	10.594594	6.531203	8.473586
H	8.208455	5.773693	10.216263
H	8.527971	5.450698	8.499058
H	9.347441	3.424937	9.608860
H	10.794672	4.516382	9.635080

H	9.671652	4.356591	11.073702
H	12.750740	13.271586	8.249379
H	10.343057	6.642760	11.474426
H	11.604934	5.845538	10.518129
H	11.522158	7.638643	10.698602
H	5.582178	10.290019	13.491394
H	4.771967	7.964309	6.709154

Conversions from L- to D- Form

MC:

S	12.221373	11.095945	12.764386
S	8.778756	12.727444	6.612382
O	7.388719	6.671596	11.550782
O	11.389550	12.921615	9.517931
O	11.989129	8.131392	10.976546
O	10.388409	6.130376	11.591580
N	9.421500	12.162210	10.226282
N	10.253917	9.595277	10.635329
N	8.005428	8.756263	10.888708
C	14.008760	11.182214	12.816678
C	9.283662	14.183301	5.758024
C	10.751992	11.964272	9.953254
C	8.484213	11.130872	10.388578
C	8.855211	9.789036	10.553102
C	11.078824	10.462042	9.739956
C	8.427873	7.620107	11.328094
C	9.856823	7.437261	11.859748
C	10.804299	8.398993	11.080535
C	6.967957	6.742844	12.892116

C	7.674181	7.198983	13.982061
C	9.030134	7.644366	14.227614
C	10.045670	7.738952	13.333622
C	11.007106	10.279202	8.161299
C	11.030450	8.822001	7.785314
C	12.273405	8.247870	7.346352
C	12.363420	6.867770	7.289745
C	11.251168	6.064023	7.483356
C	10.060798	6.630052	7.955297
C	9.928688	8.032847	8.095347
C	7.067912	11.527963	10.282720
C	6.356966	11.621747	11.659172
C	7.288193	12.381874	12.714722
C	6.352106	10.815051	9.169298
H	14.373303	10.166983	12.533369
H	14.164721	11.327779	13.857043
H	8.542932	14.949180	5.801355
H	9.340686	13.733624	4.731265
H	8.782931	13.120115	7.929661
H	9.076390	13.159203	10.110864
H	12.057912	10.328124	10.022816
H	5.901134	6.510634	12.934825
H	7.043401	7.249924	14.898129
H	9.195563	7.825345	15.268734
H	11.075486	7.992203	13.613528
H	10.128016	10.728992	7.773952
H	11.853068	10.800557	7.774200
H	13.112722	8.856361	7.155387
H	13.330567	6.443326	7.098936

H	11.318724	5.050820	7.182735
H	9.213496	5.916281	8.076664
H	8.939362	8.459588	8.401196
H	7.184134	12.584348	9.903203
H	5.485347	12.260526	11.471832
H	6.025230	10.619846	12.026296
H	7.825663	13.217890	12.188259
H	6.662536	12.872112	13.513667
H	8.010676	11.642753	13.219286
H	11.355819	6.267838	11.493505
H	5.422369	11.341509	8.863783
H	7.035218	10.763983	8.295714
H	6.122699	9.777121	9.445385
H	14.420985	11.976673	12.139812
H	10.222898	14.524446	6.255032

TS1:

S	12.210835	11.329114	12.389952
S	10.054069	11.993369	6.008055
O	7.461906	6.709246	11.466829
O	11.458779	12.709810	8.833340
O	11.878696	8.238700	11.328229
O	10.143141	6.521759	12.229630
N	9.475890	12.053830	9.801816
N	10.216059	9.621991	10.436749
N	8.003006	8.795879	10.570317
C	13.933495	10.666648	12.375488
C	10.161568	13.844103	5.997727
C	10.764170	11.742685	9.420743

C	8.472842	11.107520	10.069165
C	8.832070	9.790834	10.266261
C	11.224453	10.450638	9.749496
C	8.379884	7.784636	11.240380
C	9.633864	7.831784	12.012091
C	10.681876	8.584486	11.100328
C	6.734209	6.971797	12.619374
C	7.071855	7.721854	13.678926
C	8.293682	8.375280	14.075899
C	9.410725	8.533497	13.333188
C	12.219741	9.831002	8.769642
C	11.693980	8.655209	8.099447
C	12.483119	7.489768	7.977112
C	11.989032	6.336604	7.328942
C	10.693709	6.330597	6.716910
C	9.973700	7.567477	6.644080
C	10.488405	8.640912	7.366255
C	7.012053	11.553483	10.049935
C	6.369648	11.479403	11.408066
C	7.205428	11.803309	12.735454
C	6.301764	10.856165	8.862592
H	14.137550	9.669911	12.031778
H	14.286115	10.777646	13.408595
H	9.471321	14.183418	6.740641
H	9.853127	14.081924	4.934001
H	10.864743	11.927391	7.135309
H	9.148996	13.014704	9.644979
H	11.944654	10.857584	10.833115
H	5.822147	6.442221	12.446118

H	6.348546	7.695642	14.470111
H	8.252815	8.775259	15.065998
H	10.235067	9.128712	13.707131
H	12.374203	10.779910	8.191339
H	13.131273	9.624945	9.312103
H	13.506965	7.677022	8.360747
H	12.616987	5.528817	7.240738
H	10.215568	5.471581	6.225358
H	9.068533	7.687448	6.083517
H	9.888980	9.572076	7.328249
H	7.014947	12.612080	9.766032
H	5.466210	12.086912	11.430626
H	6.030672	10.446894	11.635406
H	7.573988	12.847064	12.885408
H	6.647912	11.511834	13.676281
H	8.077740	11.177821	12.686349
H	11.087028	6.705975	12.072939
H	5.325477	11.197470	8.624178
H	6.948738	11.125730	7.983622
H	6.297553	9.767654	8.994882
H	14.470471	11.306819	11.681801
H	11.162072	14.339983	6.257396

INT:

S	12.089348	11.454423	13.698445
S	10.554478	12.136389	6.644337
O	7.287409	6.556900	10.643648
O	11.850605	12.482694	10.266896
O	11.453809	8.071089	12.245107

O	9.777360	6.016972	11.889897
N	9.630920	12.008371	10.758722
N	10.100214	9.373617	10.915989
N	7.795051	8.785973	10.503839
C	13.871328	11.114330	13.552208
C	10.582518	13.943193	6.441654
C	10.990790	11.587087	10.444006
C	8.558966	11.135804	10.459147
C	8.725817	9.767570	10.681962
C	11.186072	10.205667	10.493266
C	8.091535	7.616356	10.925075
C	9.201759	7.307189	11.976340
C	10.367041	8.368727	11.751875
C	6.502224	5.999910	11.608377
C	6.484811	6.392593	12.907966
C	7.351172	7.267653	13.636921
C	8.570494	7.655976	13.309820
C	12.355944	9.589404	9.892190
C	11.924871	8.451626	8.996853
C	12.371861	7.124083	9.034122
C	11.735368	6.162294	8.219552
C	10.796353	6.548925	7.268960
C	10.403304	7.853067	7.187910
C	10.927175	8.822309	8.078496
C	7.261636	11.880005	10.134078
C	6.435130	11.985669	11.430887
C	7.223133	12.554411	12.613342
C	6.410002	11.306192	8.992077
H	14.024403	10.166473	13.090887

H	14.305985	11.153892	14.559278
H	9.615673	14.272419	6.714415
H	10.724002	14.189997	5.440721
H	10.360245	12.173863	8.002798
H	9.443880	12.973985	10.612130
H	11.794036	11.068245	12.348886
H	5.816922	5.223882	11.195875
H	5.753204	5.941856	13.607075
H	6.903983	7.607436	14.551555
H	9.110000	8.361752	13.950395
H	12.823998	10.377390	9.252206
H	13.096898	9.301683	10.669668
H	13.152009	6.807547	9.769756
H	11.976954	5.150113	8.297757
H	10.369996	5.858117	6.610057
H	9.654276	8.160879	6.448997
H	10.568683	9.844213	8.139107
H	7.600528	12.905772	9.761564
H	5.470508	12.527129	11.368645
H	6.165587	10.946449	11.612120
H	7.778859	13.456234	12.252994
H	6.499676	12.963387	13.353406
H	7.928113	11.877710	13.095490
H	10.667480	6.187955	12.263413
H	5.584654	11.978017	8.690028
H	6.963785	11.016319	8.059700
H	5.928770	10.425261	9.415941
H	14.348258	11.906091	12.931875
H	11.416902	14.600217	6.896989

TS2:

S	12.117147	11.770205	13.133115
S	10.365676	11.828465	6.111718
O	6.694922	7.803367	10.571838
O	11.962781	13.126261	9.557117
O	11.325568	8.707829	11.153694
O	9.204842	7.128286	11.453444
N	9.745903	12.749372	10.078724
N	9.966101	10.100360	9.881256
N	7.567835	9.727719	9.788248
C	13.995551	11.596291	12.936057
C	10.068111	13.548653	6.437809
C	11.000788	12.314095	9.611280
C	8.587725	11.979327	9.996155
C	8.629335	10.642328	9.826161
C	10.935902	10.888131	9.039664
C	7.775772	8.691927	10.529429
C	8.964601	8.505821	11.381285
C	10.229987	9.109451	10.814941
C	6.340509	7.303706	11.788495
C	6.579694	7.770842	13.049710
C	7.474754	8.825718	13.482720
C	8.555394	9.165193	12.712146
C	12.260716	10.239944	8.899717
C	12.150464	8.916613	8.094473
C	13.337420	8.094279	7.836598
C	13.140935	6.833258	7.230230
C	11.807973	6.512990	6.827007

C	10.701006	7.339620	7.010524
C	10.875685	8.574759	7.594141
C	7.334896	12.791115	10.144725
C	6.660842	12.896207	11.519680
C	7.580752	13.329657	12.659636
C	6.181851	12.349280	9.182823
H	14.239532	10.567273	12.676716
H	14.461988	11.852739	13.909691
H	9.288076	13.767030	7.181482
H	9.735889	14.094244	5.538809
H	10.287061	11.300902	7.666595
H	9.692744	13.789107	10.067826
H	11.802536	10.519050	12.746748
H	5.587421	6.543985	11.606430
H	5.778003	7.451012	13.708552
H	7.342247	9.332675	14.461190
H	9.147941	10.042005	12.896082
H	12.798320	10.933273	8.236688
H	12.772013	10.070330	9.846273
H	14.351336	8.476350	7.977842
H	13.965071	6.161885	6.913667
H	11.597637	5.574012	6.358517
H	9.772475	6.954912	6.566625
H	10.009501	9.252609	7.714016
H	7.511198	13.812467	9.710292
H	5.718750	13.438803	11.384560
H	6.318468	11.870873	11.698817
H	8.163718	14.262882	12.501852
H	6.959363	13.432135	13.576837

H	8.330605	12.542053	12.785574
H	10.073120	6.999276	11.863249
H	5.333016	13.016213	9.253254
H	6.452036	12.248100	8.128116
H	5.803573	11.373253	9.524715
H	14.482097	12.307983	12.235476
H	10.996317	14.184402	6.653430

P:

S	12.111076	11.398144	13.550555
S	10.308867	12.248527	6.432803
O	7.145332	7.145882	11.132711
O	11.759516	13.109405	9.807864
O	11.734482	8.525640	11.544305
O	9.708467	6.896091	12.444736
N	9.542330	12.509736	9.982415
N	10.175457	9.915029	10.474212
N	7.816857	9.260124	10.323437
C	13.897874	11.042686	13.310976
C	10.511344	14.011262	6.119086
C	10.915338	12.197941	9.781281
C	8.495687	11.576966	10.130828
C	8.783994	10.266714	10.313466
C	11.074415	10.693492	9.496712
C	8.092321	8.217531	11.035689
C	9.295169	8.210384	12.024613
C	10.577641	8.951245	11.284801
C	6.345813	7.232669	12.260655
C	6.592555	7.973685	13.356654

C	7.751530	8.665090	13.887772
C	8.960756	8.859900	13.338299
C	12.418980	10.146083	9.209723
C	12.525490	8.815841	8.508661
C	13.704206	8.070179	8.463530
C	13.734136	6.754461	7.926511
C	12.617847	6.197974	7.416329
C	11.451826	6.961791	7.401195
C	11.383140	8.242402	7.883181
C	7.112322	12.127993	9.981435
C	6.260238	11.867550	11.214588
C	6.989202	12.162948	12.499063
C	6.358356	11.547696	8.741039
H	13.981632	10.111446	12.696824
H	14.291427	10.835767	14.325642
H	9.629395	14.625903	6.374859
H	10.816717	14.149690	5.074932
H	10.348074	10.550190	8.660542
H	9.290062	13.496630	10.134191
H	11.634097	11.004772	12.399490
H	5.484561	6.627265	12.024144
H	5.777566	7.921327	14.082092
H	7.567770	8.956961	14.925067
H	9.709036	9.446308	13.855440
H	12.806758	10.906242	8.511378
H	13.108945	10.148531	10.074034
H	14.562706	8.461845	8.963681
H	14.670877	6.225036	7.979105
H	12.685954	5.242393	6.884978

H	10.559981	6.503229	7.000972
H	10.426603	8.704539	7.758725
H	7.348477	13.180148	9.801374
H	5.329123	12.431715	11.148204
H	6.005189	10.812138	11.050565
H	7.426372	13.155394	12.551902
H	6.282537	12.080038	13.317245
H	7.840302	11.497807	12.656549
H	10.267815	6.504056	11.704808
H	5.383831	12.019381	8.527773
H	7.049953	11.779243	7.962132
H	6.251428	10.452780	8.804472
H	14.410506	11.868219	12.831233
H	11.337703	14.552091	6.654804
